# Heterogeneous antibodies against SARS-CoV-2 spike receptor binding domain and nucleocapsid with implications for COVID-19 immunity

**DOI:** 10.1172/jci.insight.142386

**Published:** 2020-09-17

**Authors:** Kathleen M. McAndrews, Dara P. Dowlatshahi, Jianli Dai, Lisa M. Becker, Janine Hensel, Laura M. Snowden, Jennifer M. Leveille, Michael R. Brunner, Kylie W. Holden, Nikolas S. Hopkins, Alexandria M. Harris, Jerusha Kumpati, Michael A. Whitt, J. Jack Lee, Luis L. Ostrosky-Zeichner, Ramesha Papanna, Valerie S. LeBleu, James P. Allison, Raghu Kalluri

**Affiliations:** 1 Metastasis Research Center, Department of Cancer Biology, University of Texas MD Anderson Cancer Center, Houston, Texas, USA.; 2 Department of Microbiology, Immunology and Biochemistry, University of Tennessee Health Science Center College of Medicine, Memphis, Tennessee, USA.; 3 Department of Biostatistics, University of Texas MD Anderson Cancer Center, Houston, Texas, USA.; 4 Division of Infectious Diseases, Department of Internal Medicine, and; 5 The Fetal Center, Department of Obstetrics, Gynecology and Reproductive Sciences, University of Texas McGovern Medical School at Houston, Houston, Texas, USA.; 6 Feinberg School of Medicine, Northwestern University, Chicago, Illinois, USA.; 7 Department of Immunology, University of Texas MD Anderson Cancer Center, Houston, Texas, USA.; 8 Department of Bioengineering, Rice University, Houston, Texas, USA.; 9 Department of Molecular and Cellular Biology, Baylor College of Medicine, Houston, Texas, USA.

**Keywords:** COVID-19, Immunology, Adaptive immunity

## Abstract

Evaluation of potential immunity against the novel severe acute respiratory syndrome (SARS) coronavirus that emerged in 2019 (SARS-CoV-2) is essential for health, as well as social and economic recovery. Generation of antibody response to SARS-CoV-2 (seroconversion) may inform on acquired immunity from prior exposure, and antibodies against the SARS-CoV-2 spike protein receptor binding domain (S-RBD) are speculated to neutralize virus infection. Some serology assays rely solely on SARS-CoV-2 nucleocapsid protein (N-protein) as the antibody detection antigen; however, whether such immune responses correlate with S-RBD response and COVID-19 immunity remains unknown. Here, we generated a quantitative serological ELISA using recombinant S-RBD and N-protein for the detection of circulating antibodies in 138 serial serum samples from 30 reverse transcription PCR–confirmed, SARS-CoV-2–hospitalized patients, as well as 464 healthy and non–COVID-19 serum samples that were collected between June 2017 and June 2020. Quantitative detection of IgG antibodies against the 2 different viral proteins showed a moderate correlation. Antibodies against N-protein were detected at a rate of 3.6% in healthy and non–COVID-19 sera collected during the pandemic in 2020, whereas 1.9% of these sera were positive for S-RBD. Approximately 86% of individuals positive for S-RBD–binding antibodies exhibited neutralizing capacity, but only 74% of N-protein–positive individuals exhibited neutralizing capacity. Collectively, our studies show that detection of N-protein–binding antibodies does not always correlate with presence of S-RBD–neutralizing antibodies and caution against the extensive use of N-protein–based serology testing for determination of potential COVID-19 immunity.

## Abstract 2

Detection of SARS-CoV-2 nucleocapsid protein binding antibodies does not always correlate with the presence of spike protein receptor binding domain neutralizing antibodies.

## Introduction

COVID-19, a respiratory illness caused by infection with the novel coronavirus SARS-CoV-2, is a rampant health crisis that has severely affected the financial security and access to care of many, in particular our most vulnerable communities (1, 2). The activation of the immune system in response to SARS-CoV-2 infection and the clinical sequela is complex, and further studies are required to measure precise immune responses and development of immunity. To this end, the development of serological assays to quantify circulating antibodies against SARS-CoV-2 are actively pursued in hope that such tests would inform on prior exposure and possibly immunity against the virus (3). Reports on immunity (detection of antibodies) against coronaviruses (mainly SARS-CoV) acquired from exposure indicate circulating antibodies are observed at 2 years and beyond following recovery (4, 5).

It remains unclear as of today what percentage of the population has been exposed to SARS-CoV-2 and remained asymptomatic, or mildly symptomatic, because they did not require care and thus were not captured in health care records. Emerging data indicate that these unaccounted cases could underestimate the reported percentage of the population that has been exposed to, and possibly developed immunity to, SARS-CoV-2 (6–9).

The detection of circulating antibodies against SARS-CoV-2 may inform on immunity to the virus, and ongoing efforts toward sensitive and specific assays include the development of lateral flow chromatographic immunoassay or ELISA. The receptor binding domain of spike protein (S-RBD) emerged as a potential antigen against which humoral immunity may develop, and the role of S-RBD in viral entry suggests antibodies against these proteins may present with neutralizing properties and immunity to COVID-19; recent studies have suggested such a possibility (10–15).

While seroconversion yielding circulating IgG antibodies against S-RBD may inform on acquired SARS-CoV-2 immunity (vide supra), many commercial serology detection assay kits used by several health care providers and vendors detect binding antibodies against N-protein to establish seroconversion after potential SARS-CoV-2 infection (16, 17). As of August 5, 2020, Abbott has shipped over 13 million serological tests and Roche is expected to produce 10+ million tests for use in the United States, indicating these tests are widely clinically used (16, 17). In contrast, the majority of published studies on SARS-CoV-2 seroconversion have focused on full-length S-protein and S-RBD (10, 18–21). Whether the presence of antibodies against nucleocapsid protein (N-protein) correlates to having antibodies against S-RBD and the capacity for neutralization to confer potential immunity remains unknown. Therefore, this study was designed to (a) measure the levels of binding and neutralizing antibodies against S-RBD and N-protein of SARS-CoV-2 in 602 serum samples from COVID-19 intensive care unit (ICU) patients and healthy/non–COVID-19 samples, (b) determine whether quantitative S-RBD antibody response informs the clinical course of ICU-admitted COVID-19 patients, (c) investigate whether detection of binding antibodies against N-protein correlates with detection of binding antibodies against S-RBD, and (d) evaluate whether individuals with N-protein–binding antibodies exhibit SARS-CoV-2 neutralization capacity associated with S-RBD.

## Results

### Production of SARS-CoV-2 recombinant proteins.

The SARS-CoV-2 surface glycoprotein, termed spike protein, is composed of 2 subunits (S1 and S2), with the S1 subunit containing the RBD (Supplemental Figure 1A; supplemental material available online with this article; https://doi.org/10.1172/jci.insight.142386DS1). The S-RBD was expressed in FreeStyle 293-F (293F) cells using a plasmid that was validated using targeted digestion with restriction enzymes and DNA sequencing (Supplemental Figure 1, B–D, and full, uncut gels). The incorporated His-tag was used for subsequent purification from conditioned media of transfected cells. The N-protein was expressed in 293T/17 cells (Supplemental Figure 1, E–G, and full, uncut gels). The purified S-RBD was validated using Western blot for immunodetection of the His-tag and the RBD using an RBD-specific antibody (Supplemental Figure 2, A–C, and full, uncut gels). N-protein was evaluated by SDS-PAGE and Western blot analysis for the immunodetection of the N-protein (Supplemental Figure 2D, and full, uncut gels).

### Serology assays (ELISA) for the detection of antibodies against SARS-CoV-2 proteins.

**Figure 1:**
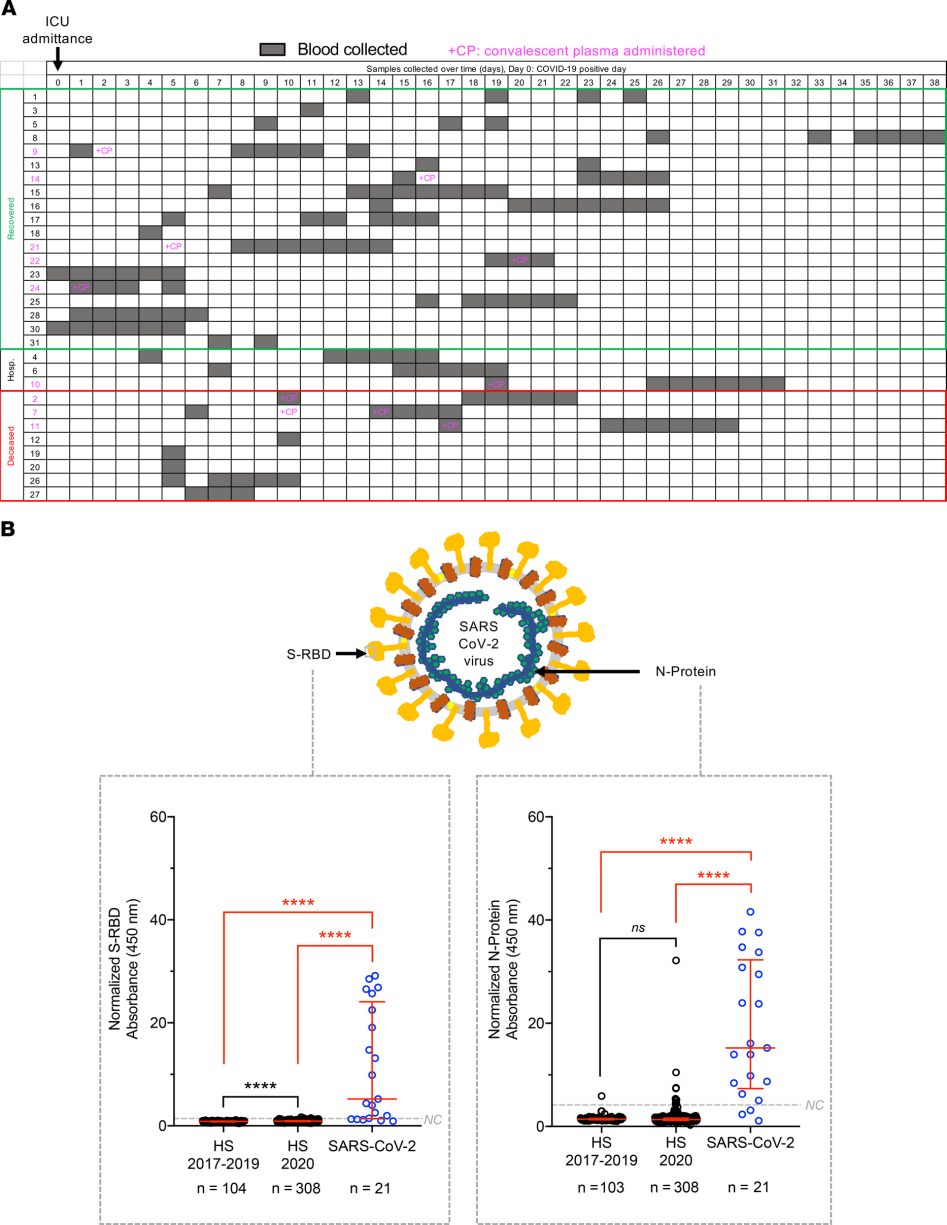
Detection of serum binding antibodies against SARS-CoV-2 proteins in patients with PCR-confirmed COVID-19 and healthy samples. (**A**) Timeline of COVID-19 diagnosis/ICU admittance, serum sample collection, and convalescent plasma (CP) administration. Time 0 is defined as day of COVID-19 diagnosis (PCR positive for SARS-CoV-2) and ICU admittance. Blood collections are denoted in gray and CP administration is denoted in pink. Patients were stratified based on current status (recovered, hospitalized, or deceased). Patient 29 from our cohort had symptoms but was PCR negative for SARS-CoV-2; this sample was not included in figures since there was no proof of disease. (**B**) Schematic of SARS-CoV-2 viral structure (top panel) and antigens assayed (bottom panel). S-protein, light orange; envelope protein, yellow; membrane glycoprotein, dark orange; RNA, blue; N-protein, green. Absorbance normalized to the respective no antigen control for each sample at 450 nm plotted for S-RBD (left panel), and N-protein (right panel), antigen coating with the most recent (or only) SARS-CoV-2 samples not treated with CP (*n* = 21) and healthy samples collected in 2017–2019 (HS 2017–2019, *n* = 104 for S-RBD, *n* = 103 for N-protein) and 2020 (HS 2020, *n* = 308). Data are presented with each dot representing the mean normalized absorbance for a given serum sample; the red bar depicts the median ± interquartile range of all samples. HS, healthy sample; NC (line), negative control cutoff (see Methods). Kruskal-Wallis with Dunn’s multiple-comparisons test performed. ****P < 0.0001; *ns*, not significant.

Purified viral proteins were used to coat the ELISA plates (Supplemental Figure 3A; see Methods). The performance of the purified protein in ELISA was first evaluated using anti–S-RBD antibodies. Anti-RBD antibodies specifically detected the recombinant S-RBD (Supplemental Figure 3B). Next, we evaluated the seroconversion of patients admitted to the ICU after approximately 5 to 7 days of viral symptoms and SARS-CoV-2 infection confirmed by reverse transcription PCR (RT-PCR) at time of admittance (Figure 1A and Table 1). Multiple serum samples (*n* = 138) following RT-PCR confirmation of COVID-19–positive status were obtained for at least 11 days after onset of symptoms (Figure 1A and Table 1). Among the 30 patients studied in the span of 2 months, 19 patients recovered and were discharged, 3 patients remained hospitalized, and 8 patients died (Figure 1A and Table 1). Nine patients received CP early in their clinical course while hospitalized (Figure 1A and Table 1). Patient characteristics such as age, sex, comorbidities, complications, and therapies are listed in Table 1. Additionally, we also analyzed 412 serum samples from healthy individuals, collected from June 2017 to December 2019 (*n* = 104) and January to June 2020 (*n* = 308), and 52 serum samples from non–COVID-19 individuals collected in a hospital setting (May 2020). For patients who were administered CP, it is not possible to distinguish exogenous and endogenous antibodies; thus, we excluded these samples from analysis and found a significant increase in both S-RBD and N-protein signal for SARS-CoV-2 samples compared with healthy samples collected between 2017–2019 and 2020 (Figure 1B). Similar trends were observed when samples from patients treated with CP were included (Supplemental Figure 4, A and B). Moreover, power analysis revealed these analyses were sufficiently powered regardless of inclusion or exclusion of CP-treated samples (Supplemental Figure 4C).

**Figure 2:**
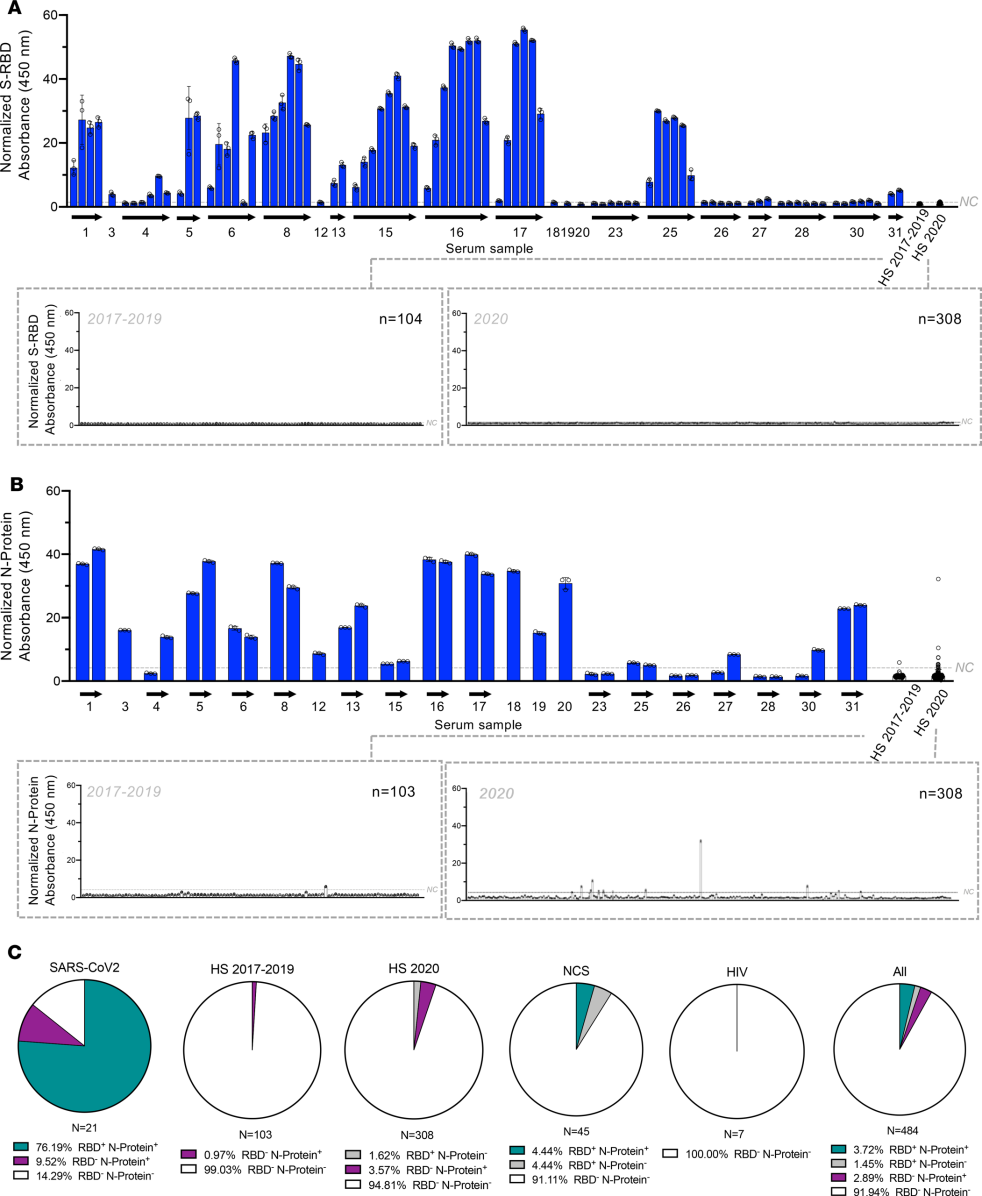
Comparison of seroconversion in patients with COVID-19 and healthy individuals. (**A**) ELISA with S-RBD protein coating and 1:100 dilution of repeated serum samples of patients with SARS-CoV-2 and healthy individuals. Absorbance normalized to the respective no antigen control for each sample at 450 nm reported. SARS-CoV-2 (blue), *n* = 88 (from 21 patients); HS 2017–2019 (white), *n* = 104; HS 2020 (white), *n* = 308. Arrows list consecutive serum samples evaluated for each case. Inset graphs depict the data separated based on healthy serum collected from 2017 to 2019 (left inset) and 2020 (right inset). (**B**) ELISA with N-protein coating and 1:100 dilution of the first and last serum samples of patients with SARS-CoV-2 and healthy individuals. Absorbance normalized to the respective no antigen control for each sample at 450 nm reported. SARS-CoV-2 (blue), *n* = 37 (from 21 patients); HS 2017–2019 (white), *n* = 103; HS 2020 (white), *n* = 308. Arrows list consecutive serum samples evaluated for each case. Inset graphs depict the data separated based on healthy serum collected from 2017 to 2019 (top inset) and 2020 (bottom inset). (**C**) Pie charts depicting percentage of samples positive for indicated antigens. SARS-CoV-2, *n* = 21; HS 2017–2019, *n* = 103; HS 2020, *n* = 308; non–COVID-19 samples (NCSs), *n* = 45; HIV, *n* = 7; all, *n* = 484.

Serial dilutions of serum were used to assess binding to the S-RBD–coated ELISA plates. A 1:100 or 1:20 serum dilution revealed detection of antibodies in 16 out of 21 patients with SARS-CoV-2 (Figure 2A, Supplemental Figure 4D, and Supplemental Figure 5). It is important to note that this is an analysis of patients who were admitted to the ICU with severe COVID-19 (Table 1). IgM antibodies were also evaluated in healthy samples and COVID-19 patients, and signal indicative of circulating IgM antibodies was detected in some COVID-19 cases with low IgG antibody levels, possibly indicating early stages of humoral response in these patients (Supplemental Figure 6). IgM antibodies were not detected in healthy samples collected from 2017 to 2019, with 1 sample collected in 2020 showing a weak positive signal for IgM but no IgG detection (Supplemental Figure 6). Next, we used recombinant S-RBD generated using a different plasmid (S-RBD1) to validate the findings with S-RBD. Binding antibodies were detected specifically in SARS-CoV-2 samples using both antigens (Supplemental Figure 7, A and B). In contrast, 0% of prepandemic (2017–2019, *n* = 0 out of 104) healthy control samples and 1.62% of healthy control serum samples collected in 2020 (*n* = 5 out of 308), during the COVID-19 pandemic, were positive for S-RBD (Figure 2, A and C; and Supplemental Figure 9A).

Next, we performed ELISAs using SARS-CoV-2 N-protein and compared these data to the data generated using the S-RBD. A 1:100 or 1:20 serum dilution showed detection of antibodies in 18 patients (Figure 2B, Supplemental Figure 4E, and Supplemental Figure 8). Approximately 1% of healthy serum samples collected in 2017–2019 (*n* = 1 out of 103) and 3.6% of HSs collected in 2020 (*n* = 11 out of 308) were positive for antibodies against N-protein (Figure 2, B and C; and Supplemental Figure 9B). Binding antibodies for S-RBD and N-protein were also assessed in 45 non–COVID-19 serum samples collected in a hospital setting (NCSs). The results indicate that while most samples were negative for antibodies against both SARS-CoV-2 antigens, approximately 4% of samples showed positive signal for S-RBD and N-protein (*n* = 2 out of 45), and 4% of samples showed exclusive positivity for S-RBD (*n* = 2 out of 45, Figure 2C and Supplemental Figure 10). Sera from HIV-positive patients (*n* = 7) were also evaluated because the FDA requires SARS-CoV-2 serology tests to demonstrate lack of cross-reactivity with other RNA virus–mediated diseases, including HIV (22). Binding antibodies were not detected against S-RBD or N-protein in HIV samples (Supplemental Figure 11, A and B).

**Figure 3:**
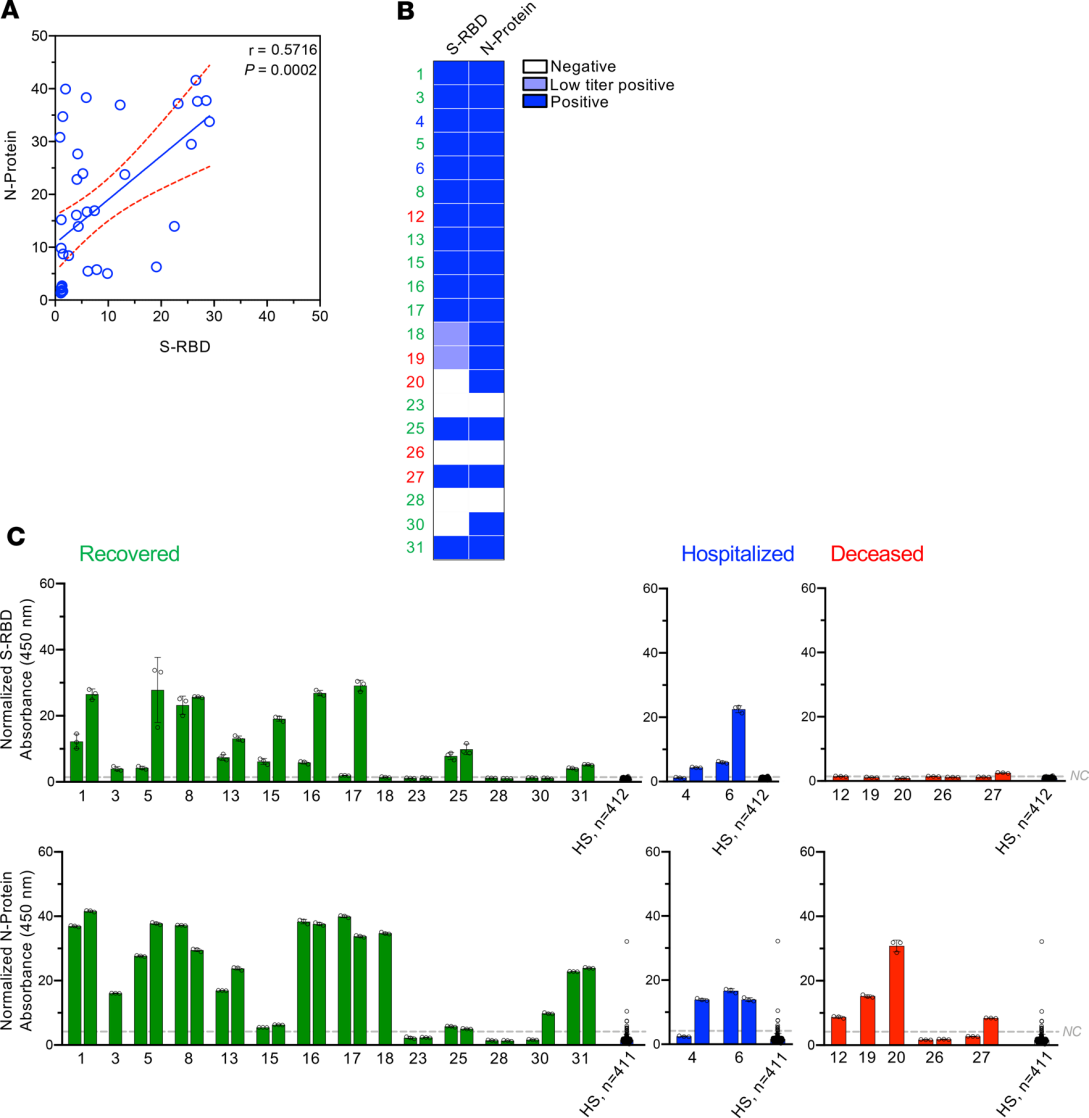
Seroconversion in patients with COVID-19 and clinical outcomes. (**A**) Correlation (Spearman’s correlation coefficient *r* displayed) contrasting the normalized A450 values from the last or only serum sample analyzed for S-RBD and N-protein. *n* = 37 serum samples (from 21 patients with SARS-CoV-2). (**B**) Heatmap depicting positive, low titer positive, or negative categorization of the last or only serum sample for each patient and for each viral protein tested. Case numbers are color-coded: green: recovered, blue: hospitalized, red: deceased. Low titer positive as defined by detecting of binding antibodies shown in Supplemental Figure 4, D and E, 1:20 titer. (**C**) Normalized S-RBD (top panels), and N-protein (bottom panels) absorbance for patients classified based on their current status (recovered, green; hospitalized, blue; deceased, red). Data are reported as mean ± standard deviation (SD) of 3 technical replicates for each sample. The first (or only) and most recent serum samples are shown. NC (line), negative control cutoff (see Methods); HS, healthy samples from 2017 to 2020 (*n* = 412 for S-RBD; *n* = 411 for N-protein).

The correlation between absorbance at 450 nm (A450) signal for N-protein and S-RBD indicated that the relative detection of antibodies for the viral proteins was only moderately concordant (Spearman’s correlation coefficient = 0.5716; Figure 3A and Supplemental Figure 12). Serological test results, categorized as negative, low titer positive, and positive based on quantitative ELISAs (see Methods), revealed heterogeneity in the detection of circulating antibodies against the viral proteins (Figure 3B). Generally, antibodies against N-protein were more prevalent (Figure 2C, Figure 3B, and Supplemental Figure 12). This may reflect a greater percentage homology of SARS-CoV-2 N-protein to other coronaviruses (Supplemental Figure 13, A and B) (10). We also evaluated the specificity and sensitivity of the ELISAs using the last sample collected from the 21 RT-PCR–confirmed SARS-CoV-2 patients not treated with CP and HSs collected from 2017 to 2019 (Supplemental Figure 14).

### Correlating seroconversion with clinical outcome and rapid recovery.

The relative positivities for antibody detection against the different viral proteins were compared in SARS-CoV-2 patients who recovered, remained hospitalized, or succumbed to COVID-19 at the time of our analysis. Results from the first and last serum sample tested are depicted in Figure 3C (when available, all serum collection analyses are depicted in Supplemental Figure 15). Two patients (out of 14) recovered without robust detection of binding antibodies against S-RBD or N-protein (patients 23 and 28, Figure 3, B and C). Among the patients who succumbed to COVID-19 (5 out of 21), patient 26, who was hospitalized for 13 days, lacked antibodies against S-RBD and N-protein (Figure 3, B and C). Interestingly, 2 out of 5 of the deceased patients did not reveal binding antibody titers against S-RBD (patients 20 and 26) (Figure 3, B and C). This should be cautiously interpreted because 3 out of 14 patients who recovered also lacked binding antibodies against S-RBD (Figure 3, B and C) and the clinical outcomes of a small number of patients were analyzed (*n* = 21).

Nine patients also received CP (*n* = 9 out of 30; Figure 1A). In such patients, it is not possible to determine whether binding antibodies detected against any of the viral proteins were endogenously generated or exogenously provided via CP infusion. Binding antibodies against S-RBD and N-protein were consistently detected in serum samples with CP infusion (Supplemental Figure 16). Regardless of presence of binding antibodies against S-RBD, 3 patients died, 1 remained hospitalized, and 5 recovered and were discharged (Supplemental Figure 16 and Supplemental Figure 17). Sample size and limited follow-up preclude us from any conclusion regarding the efficacy of CP.

Although our sample size was small (*n* = 21 SARS-CoV-2 cases), we assessed whether seroconversion was associated with length of stay in the hospital. There was an increase in the length of stay for patients who recovered compared with those who died (Supplemental Figure 18A). Length of stay moderately correlated with S-RBD but not N-protein seroconversion evaluated at the last serum sample (Supplemental Figure 18B). Studies indicated a possible correlation between the ABO blood group loci and respiratory failure associated with COVID-19 (23, 24). In our samples, we did not observe any bias with respect to ABO blood types and length of stay or clinical outcome (Supplemental Figure 18C).

### Correlating S-RBD and N-protein seroconversion with neutralizing antibodies.

**Figure 4:**
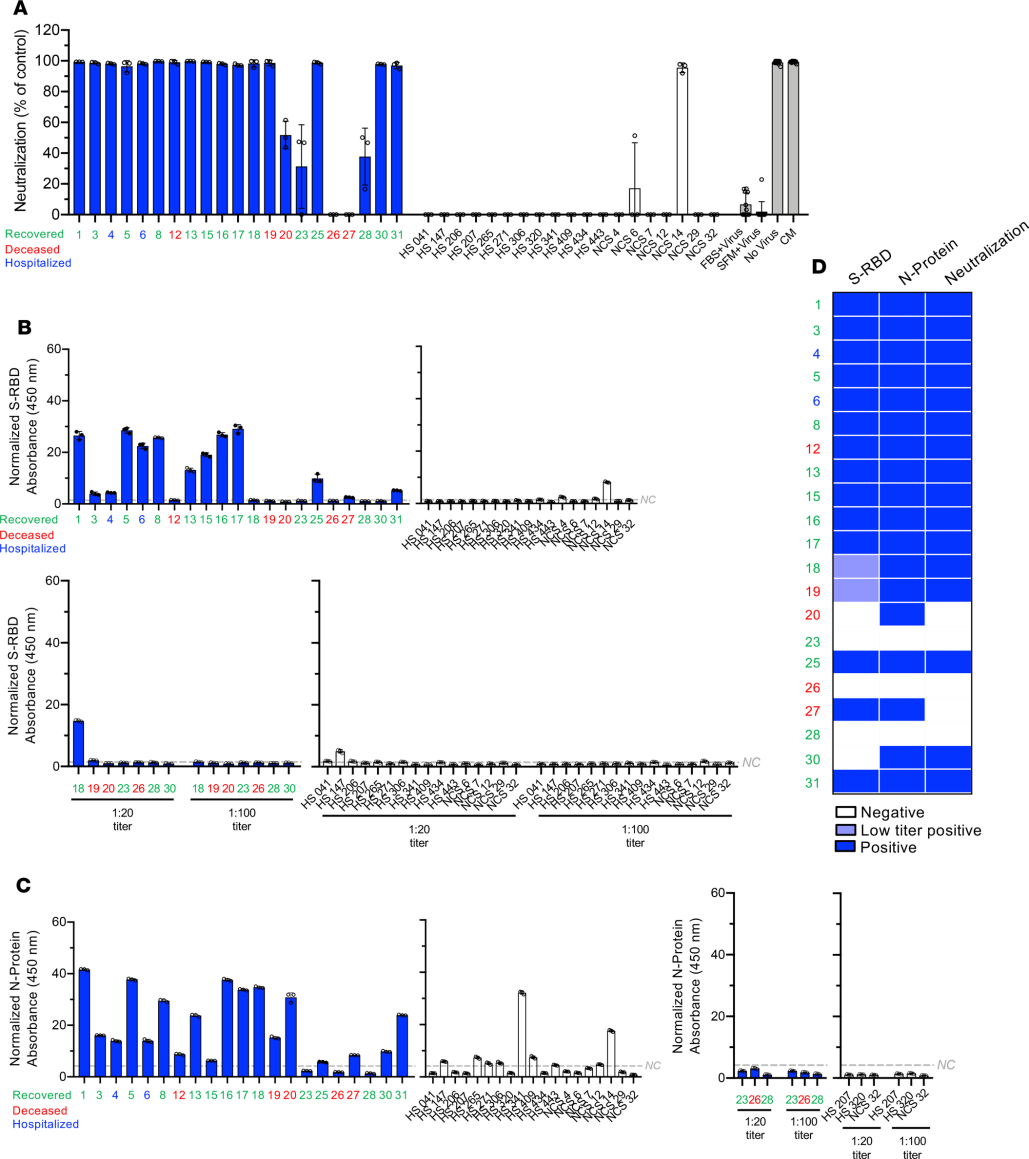
Pseudotyped SARS-CoV-2 virion neutralization activity of serum binding antibodies against S-RBD and N-protein. (**A**) Luminescence normalized to FBS+Virus control obtained from pseudovirus neutralization assay at 1:20 serum dilution. (**B**) Matched serological results for S-RBD at 1:100 serum dilution (top 2 panels) and 1:20 serum dilution (bottom 2 panels). Absorbance normalized to the respective no antigen control for each sample at 450 nm reported. Case numbers are color-coded: green: recovered, red: deceased, blue: hospitalized. (**C**) Matched serological results for N-protein at 1:100 serum dilution and 1:20 serum dilution. Absorbance normalized to the respective no antigen control for each sample at 450 nm reported. Case numbers are color-coded: green: recovered, red: deceased, blue: hospitalized. Data (**A**–**C**) are reported as mean ± standard deviation (SD) of 3 technical replicates for each sample. (**D**) Heatmap depicting positive and negative categorization of the listed serum cases for each viral protein tested in serological and neutr3alization assays. Low titer positive as defined by detecting of binding antibodies shown in Figure 2, C and D, 1:20 titer.

To determine whether seroconversion is indicative of antibodies with neutralization capacity to impart immunity, we measured serum titers capable of blocking entry of pseudovirus expressing SARS-CoV-2 S-protein (25). Briefly, the vesicular stomatitis virus (VSV) pseudovirus was engineered to contain the luciferase gene and display SARS-CoV-2 full-length S-protein on the virion membrane. Infection of Vero-TMPRSS2 cells with the pseudovirus was detected by measuring luminescence, and decreased luminescence indicated the presence of antibodies that neutralized pseudovirus infectivity (Figure 4A, Supplemental Figure 19, and Supplemental Figure 20). Detection of binding antibodies in control samples, with positivity for S-RBD or N-protein, generally did not present with neutralization function, with only 1 exception (NCS 14, Figure 4, A–C). NCS 14 exhibited neutralizing antibodies, as well as signal for both S-RBD and N-protein, at the levels of SARS-CoV-2 samples (Figure 4, A–C). Serum antibodies from HS controls with negative serology (HS 206, 207, 320; NCS 6, 7, 29, and 32) did not reveal neutralization capacity (Figure 4, A–C). In patients with SARS-CoV-2 treated with convalescent plasma (+CP), detection of binding antibodies against S-RBD and N-protein was associated with neutralization capacity (Supplemental Figure 21). Eleven patients with COVID-19 who recovered demonstrated positive serology for N-protein and S-RBD and demonstrated antibodies with neutralization capacity (Figure 4, A–D). Patient 20, who succumbed to the disease and presented with binding antibodies to just the N-protein, did not reveal neutralization capacity (Figure 4, A–D). Additionally, 2 patients who were negative for N-protein and S-RBD antibodies and recovered (patients 23 and 28) lacked neutralizing capacity (Figure 4, A–D). In patient 30, neutralization capacity was noted despite lack of binding antibodies against S-RBD (1:20, Figure 4, A–D). S-RBD antibodies were detected at low levels (1:20 titer) in 2 patients (patients 18 and 19) and exhibited neutralizing activity (Figure 4, A–D). Only 1 patient among this group recovered from COVID-19.

## Discussion

Our study demonstrates ELISA-based detection of antibodies for S-RBD and N-protein in the serum of patients infected with SARS-CoV-2. Our findings, obtained from the analysis of 138 serial serum samples from 30 SARS-CoV-2 RT-PCR–positive patients with severe disease, and 464 control serum samples collected between June 2017 and June 2020, support other studies published recently, to a certain extent (10, 18, 21, 26). While most studies have examined CP from individuals who recovered from COVID-19, we tracked 30 severely ill COVID-19 patients admitted to the ICU upon presentation to the emergency room, for at least 11 days after onset of viral symptoms. Some reports suggest that IgG levels can be detected 9 days after presentation with COVID-19 symptoms, and this study agrees with such data (10, 12, 21, 27, 28).

For S-RBD, we report no (0%) positivity in prepandemic 2017–2019 healthy serum samples and approximately 1.6% positivity in 2020 healthy serum samples. Binding antibodies were detected in 71% of severely ill COVID-19 patients at presentation and 76% by the time the last serum samples were analyzed. For the N-protein, we report approximately 1% positivity in 2017–2019 healthy serum samples and 3.57% positivity in 2020 healthy serum samples. Binding antibodies were detected in 71% of severely ill COVID-19 patients at presentation and 86% by the time the last serum samples were analyzed.

Quantitative analysis of the binding antibody response to the S-RBD and N-protein suggests that there is heterogeneous IgG response to the 2 different viral antigens. IgG seroconversion in the earliest serum samples was detected in 15 out of 21 (~71%) COVID-19 patients when N-protein or S-RBD ELISA was employed. When ELISA was performed to detect binding antibodies in the serum collected at later stages, 18 out 21 samples (~86%) were positive for N-protein, and of the N-protein–positive samples, 16 out of 18 (~89%) were also positive for S-RBD. These results suggest that developing an IgG antibody response against viral antigens is heterogeneous in nature and does not always correlate with each other. All 9 patients who received CP increased their titers against S-RBD, but only 5 increased their titers against N-protein, indicating that CP is likely associated with antibody titers against S-RBD and less so against the N-protein.

This study also suggests that presence of high-titer S-RBD–binding and –neutralizing antibodies does not lead to rapid recovery of COVID-19 patients. Additionally, 2 patients without antibodies or low-titer binding antibodies against S-RBD recovered from severe COVID-19 disease. Interestingly, serum from healthy individuals who exhibited binding antibodies against N-protein but not against the S-RBD did not exhibit neutralizing activity. These results suggest that binding antibodies against N-protein do not correlate with having S-RBD–binding antibodies or possessing neutralizing capacity. On the other hand, 94% of SARS-CoV-2 patients (*n* = 15 out of 16) with binding antibodies against S-RBD exhibited neutralizing capacity, despite low titers in some cases (1:20). These results suggest that titer of S-RBD antibodies may in some cases not be as relevant for neutralization, as compared with the affinity and avidity properties of the S-RBD–binding antibodies. In this regard, CP potency might need to be evaluated using neutralizing antibody assays rather than relying solely on titers of S-RBD–binding antibodies.

This study also brings about a caution against the use of serology tests that rely only on detection of binding antibodies against N-protein to measure potential COVID-19 immunity. In this study, approximately 3% of the healthy serum samples were positive for N-protein, while only approximately 1% of these samples were S-RBD positive. Collectively, this suggests that antibody titers against N-protein may suggest prior exposure to SARS-CoV-2 or related viruses but do not necessarily provide evidence for the presence of neutralizing antibodies. Currently, commercial companies have conducted millions of such tests using N-protein (16, 17), and antibody-positive individuals might be misled in this regard.

Careful assessments of the prognostic value of SARS-CoV-2 seroconversion and its contribution to potentially conferring immunity are urgently needed (9), and academic institutions could offer alterative avenues to commercial testing and enable the development of accurate and reliable serological testing to carefully identify individuals with neutralizing antibodies. Our study suggests that the use of a quantitative ELISA coupled with a neutralizing antibody assay can inform on efficacious CP for COVID-19 therapy and also identify individuals with acquired immunity against COVID-19.

## Methods

### Human serum.

All human serum samples (*n* = 602 analyzed for S-RBD, and *n* = 601 analyzed for N-protein) were obtained under Institutional Review Board (IRB) exemption and were deidentified. Discarded frozen sera from healthy individuals were obtained from the MD Anderson Department of Laboratory Medicine at MD Anderson Cancer Center, and donations spanned from June 2017 to May 2020 (*n* = 412). Sera from individuals collected at Memorial Hermann-Texas Medical Center (*n* = 45) in May 2020 were also analyzed and referred to as NCSs. Sera from patients with SARS-CoV-2 (*n* = 30 patients, with 138 serum samples total) and patients with HIV (*n* = 7) were also obtained from the Memorial Hermann-Texas Medical Center under the approval of the IRB at the University of Texas Health Science Center. Patient characteristics are described in Table 1. All healthy, non–COVID-19, and SARS-CoV-2 samples were heat inactivated at 56°C for 30 minutes, and HIV samples were heat inactivated at 56°C for 2 hours, in accordance with institutional biosafety and CDC guidelines. Heat inactivation has been previously shown to have negligible effects on the detection of IgG antibodies against S-RBD (18). After heat inactivation, samples were stored at –80°C until the time of assay.

### Plasmids.

Transformation of *E*. *coli* (TOP10 competent cells, Invitrogen, Thermo Fisher Scientific) cultured under ampicillin selection enabled plasmid production, which was purified using NucleoBond Xtra Maxi EF (MACHEREY-NAGEL) according to the manufacturer’s directions. Restriction digestion of the purified plasmid (Supplemental Figure 1, C and F) used enzymes purchased from New England Biolabs, and restriction digestions were carried out as recommended by the manufacturer. Digestion products were visualized following electrophoresis on a 0.7% agarose gel. The S-protein RBD-His (S-RBD1) plasmid was obtained from Addgene (plasmid 145145). N-protein-His (N-protein1) plasmid was obtained from Sino Biological (plasmid VG40588-CH).

### Cell culture and transfection.

FreeStyle 293-F cells from Thermo Fisher Scientific (R79007) were cultured in FreeStyle 293 Expression Medium (Thermo Fisher Scientific). The cells were transfected with the S-RBD-His plasmid using 293fectin reagent (Thermo Fisher Scientific) according the manufacturer’s protocol, and the cells were maintained in FreeStyle 293 Expression Medium. 293T/17 cells (ATCC) were cultured in DMEM (Corning) with 10% FBS (Gemini) and transfected with the N-protein-His plasmid with Lipofectamine 3000 (Invitrogen, Thermo Fisher Scientific) according to the manufacturer’s instructions. Conditioned media (CM) were collected from S-RBD-His–transfected cells for 2 consecutive 48-hour incubations and used for purification. Cell lysate was collected from N-protein-His–transfected cells in RIPA buffer with 1× EDTA-free protease inhibitor cocktail (Roche).

### Protein purification and quantification.

The CM from transfected 293F cells were collected, centrifuged at 800*g* for 5 minutes, filtered with a 0.22 μm filter (Thermo Fisher Scientific), and stored at –80°C before processing. Affinity purification was performed using an Akta Pure fast protein liquid chromatography system (Cytiva, formerly GE Healthcare). The S-RBD-His product (S-RBD1) purification was performed using nickel-nitrilotriacetic acid resin, where samples were supplemented to a final concentration of 30 mM imidazole (MilliporeSigma) and 1× EDTA-free protease inhibitor cocktail (Roche) before loading onto a HisTrap FF 1 mL column (Cytiva, formerly GE Healthcare), equilibrated in wash buffer (PBS 300 mM NaCl, 40 mM imidazole, pH 8.0), and eluted with a linear gradient from 40 to 500 mM imidazole. Eluted fractions were pooled, concentrated with 3000 Da (MW cutoff) ultrafiltration membrane spin column (Amicon) per the manufacturer’s instructions, dialyzed into PBS, and stored at –80°C. The purified S-RBD1 was quantified using NanoDrop OneC (280 nm absorbance, Thermo Fisher Scientific), BCA assay (Thermo Fisher Scientific) relative to bovine serum albumin (BSA) standard, and/or Qubit 3 Fluorometer (Qubit Protein Assay, Thermo Fisher Scientific) according to the manufacturer’s instructions. The reagents for the protein purifications are listed in Supplemental Table 1.

### Western blot.

S-RBD, S-RBD1, and N-protein were electrophoresed on 4%–12% SDS-PAGE gel (NOVEX) stained with SimplyBlue SafeStain (Invitrogen, Thermo Fisher Scientific). For immunolabeling, the proteins were transferred onto a 0.22 μm PVDF membrane (Bio-Rad) using a Trans-Blot Turbo Transfer System (Bio-Rad). Cell lysates were collected in RIPA buffer with protease inhibitor (Roche). To reduce proteins, DTT (final concentration 62.5 mM) was added to the 4× Laemmli sample buffer (Bio-Rad). The membranes were blocked with 5% *w/v* milk in TBS/Tween 0.1% (TBS/T) and incubated with rabbit polyclonal anti-His (catalog 2365S, Cell Signaling Technology, 1:1000), rabbit polyclonal anti–SARS-CoV-2 Spike RBD (40592-T62, Sino Biological, 1:1000), or rabbit polyclonal anti–SARS-CoV2-Nucleocapsid protein (40588-T62, Sino Biological, 1:2000) antibodies in 2% BSA TBS/T (see Supplemental Table 1). Secondary antibodies used were donkey anti-rabbit HRP (ab16284, Abcam, 1:2000) in 2% BSA TBS/T. Visualization of immunolabels was performed using West-Q Pico ECL solution (Gendepot), and chemiluminescent signals were captured using Amersham Hyperfilm (Cytiva, formerly GE Healthcare). The images were uniformly changed to gray scale.

### ELISA.

MaxiSorp C-shaped 96-well plates were coated with S-RBD, S-RBD1, or N-protein generated from plasmids from Sino Biological and Addgene. All proteins were His-tag purified. The stock S-RBD (2.5 μg/mL; 93.28 nM) was used to coat ELISA plates (Sino Biological 40592-V08H). The stock N-protein (1.25 μg/mL; 26.55 nM) was used to coat ELISA plates (Sino Biological 40588-V08B). S-RBD1 is described above. For S-RBD1, 5 μg/mL (213.68 nM) was used. For each antigen, we analyzed samples with at least 2 antigen concentrations (keeping biochemical molar equivalents in mind) and chose the antigen concentration where sufficient signal was detected. The proteins were diluted in 50 mM sodium carbonate (Na_2_CO_3_)–sodium bicarbonate (NaHCO_3_) coating buffer, pH 9.6, and allowed to bind to the well (50 microliters per well) at 37°C 2 hours or at 4°C overnight. For S-RBD1, protein was diluted in PBS, pH 7.4, and allowed to bind to the well (50 microliters per well) at 4°C overnight. The coating mixture was removed from the wells, and the wells were subsequently blocked with 5% nonfat dry milk in TBS (200 microliters per well) at room temperature for 1 hour. Serum samples were thawed on ice, serially diluted in TBS, and dispensed (50 microliters per well) in triplicates into the coated and blocked wells. For the performance assays shown in Supplemental Figure 3B, 50 μL of antibody (Supplemental Table 1) in 1% BSA was added to the wells. The plate was incubated at 37°C for 1 hour, then washed 3 times with TBS/0.1% Tween-20 (200 microliters per well). The wells were then incubated with HRP-conjugated secondary antibodies (50 microliters per well, Supplemental Table 1) at 37°C for 1 hour and washed again 3 times with TBS/0.1% Tween-20 (200 microliters per well). The last wash was removed from the well, and 3,3′,5,5′-Tetramethylbenzidine reagent (Supplemental Table 1) was added to each well (50 microliters per well). The plate was incubated at room temperature for 15 minutes and read at 650 nm using the Versamax spectrophotometer from Molecular Devices (Thermo Fisher Scientific). The reaction was stopped by adding 0.18 M sulfuric acid in each well (50 microliters per well), and the wells were read again at 450 nm. For each serum sample tested, additional wells were set up to include a no antigen control, in which no antigen was used to coat the plate, but wells were blocked with 5% milk and matching serum titers and secondary antibodies were used. The no antigen values for the given serum sample was divided from the antigen-containing wells (at matching titers) to control for nonspecific aggregation in the well despite extensive wash steps. These normalized A450 nm values are depicted in the presented graphs, with each technical replicate shown. In 4 instances, a technical replicate for a given serum sample technical triplicate was erroneous and excluded.

Positivity was determined as samples with the no antigen–normalized A450 value (see above) at 1:100 serum dilution being above or equal to the mean of normalized 2017–2019 HSs plus 5 standard deviations of that mean. Low titer positive categorization was determined as samples with the normalized OD 450 nm value being above or equal to the positivity cutoff at 1:20 serum dilution (the lowest serum dilution used for neutralization assays). The NC cutoff shown in bar graphs represents the mean plus 5 standard deviations of 2017–2019 HS.

To determine specificity and sensitivity of the ELISAs, background-normalized A450 nm values were used to define receiver operating characteristics. The chosen cutoffs and the associated specificity and sensitivities were different from those employed to define the positive and negative categorization described above.

### SARS-CoV-2/VSV pseudotype production and neutralization assays.

VSV-ΔG-luciferase pseudotypes displaying the full-length SARS-CoV-2 S-protein (Wuhan-Hu-1 strain) were generated as previously described (25) using a plasmid encoding a codon-optimized cDNA for the SARS-CoV-2 S-protein (18), which was provided by Florian Krammer at the Icahn School of Medicine at Mount Sinai (New York, New York, USA). SARS-CoV-2–specific titers were determined on Vero-E6 and VeroE6-TMPRSS2 cells, the latter of which were obtained from the Japanese Collection of Research Bioresources (1819, ref. 29). Residual infectivity from the VSV-G pseudotyped ΔG-luciferase inoculum was neutralized immediately after virus adsorption by incubation for 30 minutes with hybridoma culture supernatant containing a VSV G-specific monoclonal antibody (I1; 8G5F11; ref. 30).

SARS-CoV-2–neutralizing activity in sera was determined using VeroE6-TMPRSS2 cells. Approximately 2 × 10^3^ infectious units of SARS-CoV-2/ΔG-luciferase pseudotype virus were mixed with increasing 2-fold dilutions of patient sera that had been complement inactivated by incubation at 56°C for 30 minutes. The virus-sera admixtures were incubated at 37°C for 1 hour (*n* = 3 technical replicates) and then added directly to VeroE6-TMPRSS2 cells in a 96-well plate that had been passaged approximately 18 hours prior. Luciferase activity was assayed 17 hours postinoculation using the XTND Luc-Screen kit as per the manufacturer’s instructions (Applied Biosystems, Thermo Fisher Scientific), and relative light units were read using a Bio-Tek Synergy 2 plate reader. The neutralization assays were carried out as 4 independent experiments, each of which included positive and negative controls. Positive controls were virus in serum-free media (SFM+Virus, *n* = 3 technical replicates) and virus with FBS (FBS+Virus, *n* = 3 technical replicates). In 1 assay, 1 technical replicate was found as an outlier for the FBS+Virus control and was excluded (*n* = 2). NCs were CM or media without virus (no Virus). Data were normalized to the corresponding FBS+Virus positive control for assay and expressed as relative percentage subtracted from 100. Any percentages less than 0% were set to 0%.

### Data availability.

Source data for all figures are provided in Supplemental Table 2.

### Statistics.

Statistical analysis and generation of the graphs and heatmaps were carried out using GraphPad Prism 8.0, or a custom-written script in MATLAB 2019a. Power calculations were performed using a post hoc Wilcoxon-Mann-Whitney test in GPower. The mean and standard deviation from the mean presented in the figures, unless otherwise noted, are described in the figure legends. Kruskal-Wallis test was applied to non-normally distributed data for comparing multiple groups, with Dunn’s multiple-comparisons adjustments to account for multiplicity in testing the difference between groups. For normally distributed data, 1-way ANOVA was applied to compare multiple groups with Dunnett’s multiple-comparisons adjustments to account for multiplicity in testing the difference between groups. Spearman’s correlation coefficient was calculated for quantifying the association between 2 continuous variables. Two-sided *P* values were reported, with *P* < 0.05 considered significant.

### Study approval.

SARS-CoV-2 samples, NCSs, and HIV samples were collected with IRB review and approval at the University of Texas (UT) Health Science Center (Houston, Texas, USA). A waiver of consent was obtained because samples would otherwise be discarded, in addition to the critically ill status of and requirements to maintain distance from COVID-19–positive patients. All human serum samples were deidentified and IRB reviewed and exempted at UT MD Anderson Cancer Center (Houston, Texas, USA).

## Author contributions

RK conceptually designed the strategy for this study. KMM, DPD, JD, LMB, JH, LMS, JML, MRB, KWH, NSH, AMH, and JK performed experiments. KMM and VSL analyzed data. JJL advised on statistical analyses. MAW contributed to the development and conducting of the pseudovirus neutralization assay. LLOZ provided the HIV-positive and COVID-19 serum samples and intellectual contribution. RP provided COVID-19 serum samples and intellectual contribution. JPA participated in discussions and provided intellectual input. KMM, VSL, and RK wrote the manuscript.

## Supplementary Material

Supplemental data

Supplemental Data Set 1

## Figures and Tables

**Table 1:**
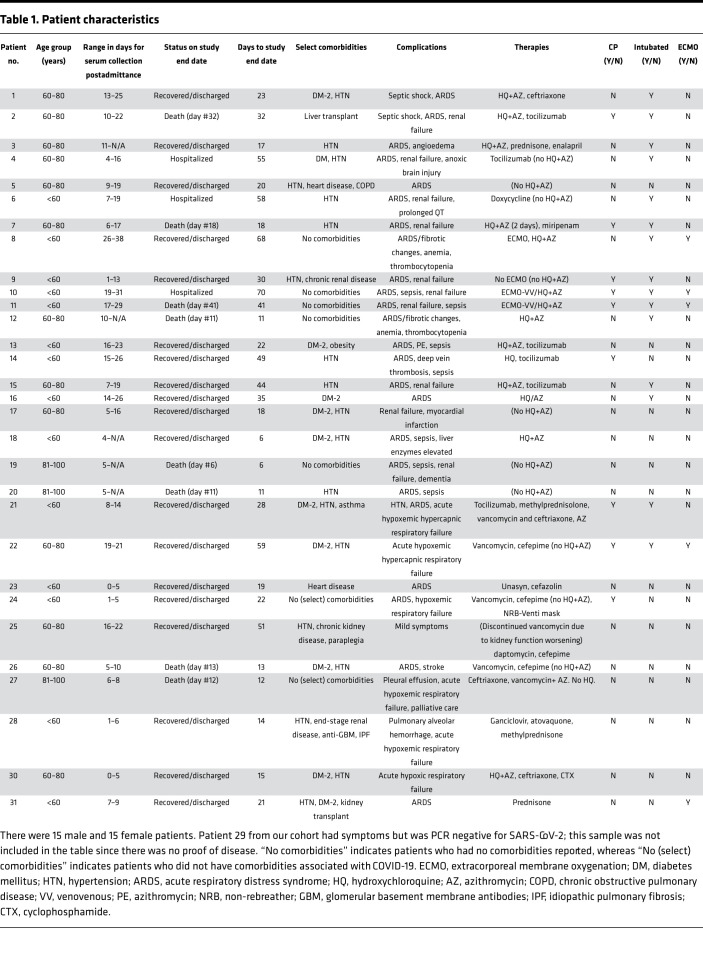
Patient characteristics

## Version 1 08/14/2020

In-Press Preview

## Version 2 09/17/2020

Electronic publication
