# Supplementary material for: Heterogeneous antibodies against SARS-CoV-2 spike receptor binding domain and nucleocapsid with implications for COVID-19 immunity
Source: JCI Insight. 2020 Sep 17;5(18):e142386. doi: 10.1172/jci.insight.142386 (PMC7526535; doi:10.1172/jci.insight.142386)

Supplementary Material

Heterogeneous antibody production against SARS–CoV–2 spike receptor binding domain and nucleocapsid protein with implications on immunity against COVID-19

Kathleen M. McAndrews[1], Dara P. Dowlatshahi[1], Jianli Dai[1], Lisa M. Becker[1], Janine Hensel[1], Laura M. Snowden[1], Jennifer M. Leveille[1], Michael R. Brunner[2], Kylie W. Holden[2], Nikolas S. Hopkins[2], Alexandria M. Harris[2], Jerusha Kumpati[2], Michael A. Whitt[2], J. Jack Lee[3], Luis L. Ostrosky-Zeichner[4], Ramesha Papanna[5], Valerie S. LeBleu[1,6], James P. Allison[7] and Raghu Kalluri[1,8,9,*]

[1]Department of Cancer Biology, Metastasis Research Center, University of Texas MD Anderson Cancer Center, Houston, TX, USA.
[2]Department of Microbiology, Immunology & Biochemistry, University of Tennessee, Memphis, TN, USA.
[3]Department of Biostatistics, University of Texas MD Anderson Cancer Center, Houston, TX,
[4]Division of Infectious Diseases, University of Texas McGovern Medical School at Houston, Houston, TX, USA.
[5]The Fetal Center, Department of Obstetrics and Gynecology, University of Texas McGovern Medical School at Houston, Houston, TX, USA.
[6]Feinberg School of Medicine, Northwestern University, Chicago, IL, USA.
[7]Department of Immunology, University of Texas MD Anderson Cancer Center, Houston, TX, USA.
[8]School of Bioengineering, Rice University, Houston, TX, USA.
[9]Department of Molecular and Cellular Biology, Baylor College of Medicine, Houston, TX, USA.

*Corresponding author
1881 East Road, Unit 1906
Houston, TX 77054
Phone number: 713-792-7780
Email: rkalluri@mdanderson.org

Figure S1

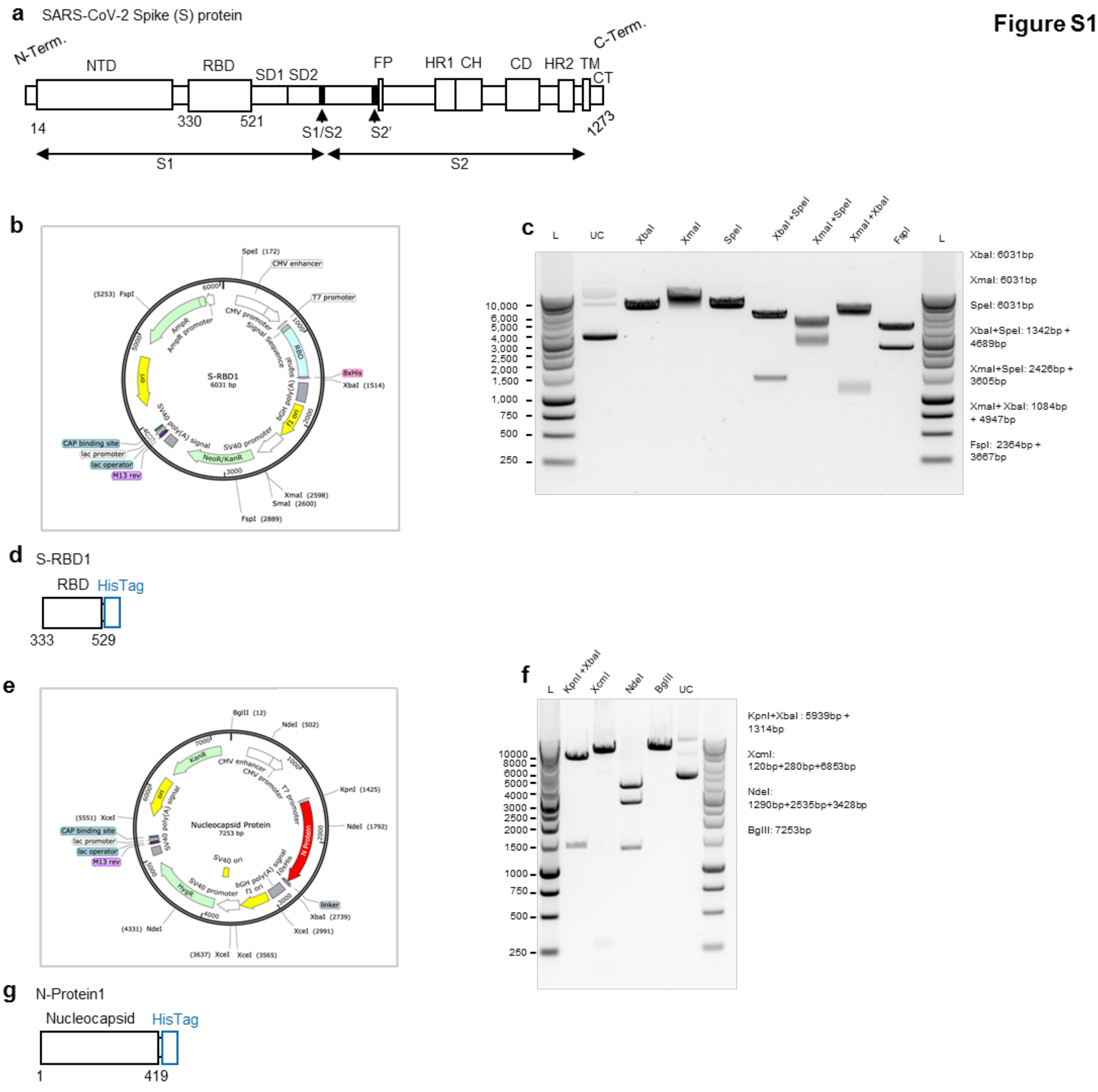

Figure S1: Plasmids used for generation of recombinant SARS-CoV-2 proteins

(**a**) Schematic of SARS-CoV-2 spike protein. NTD, N-terminal domain; RBD, receptor binding domain; S2', S2' protease cleavage site; FP, fusion peptide; HR1, heptad repeat 1; CH, central helix; CD, connector domain; HR2: heptad repeat 2; TM, transmembrane domain; CT, C-terminus. (**b**) Map of SARS-CoV-2 spike protein RBD expression plasmid construct (S-RBD1). (**c**) Agarose gel electrophoresis of restriction digested S-RBD1 plasmid. L: ladder, UC: uncut plasmid. Predicted sizes of each digestion denoted. (**d**) Schematic of S-RBD1 protein. (**e**) Map of SARS-

CoV-2 nucleocapsid protein expression plasmid construct (N-protein1). (**f**) Agarose gel electrophoresis of restriction digested N-protein1 plasmid. L: ladder, UC: uncut plasmid. Predicted sizes of each digestion denoted. (**g**) Schematic of N-protein1.

Figure S2

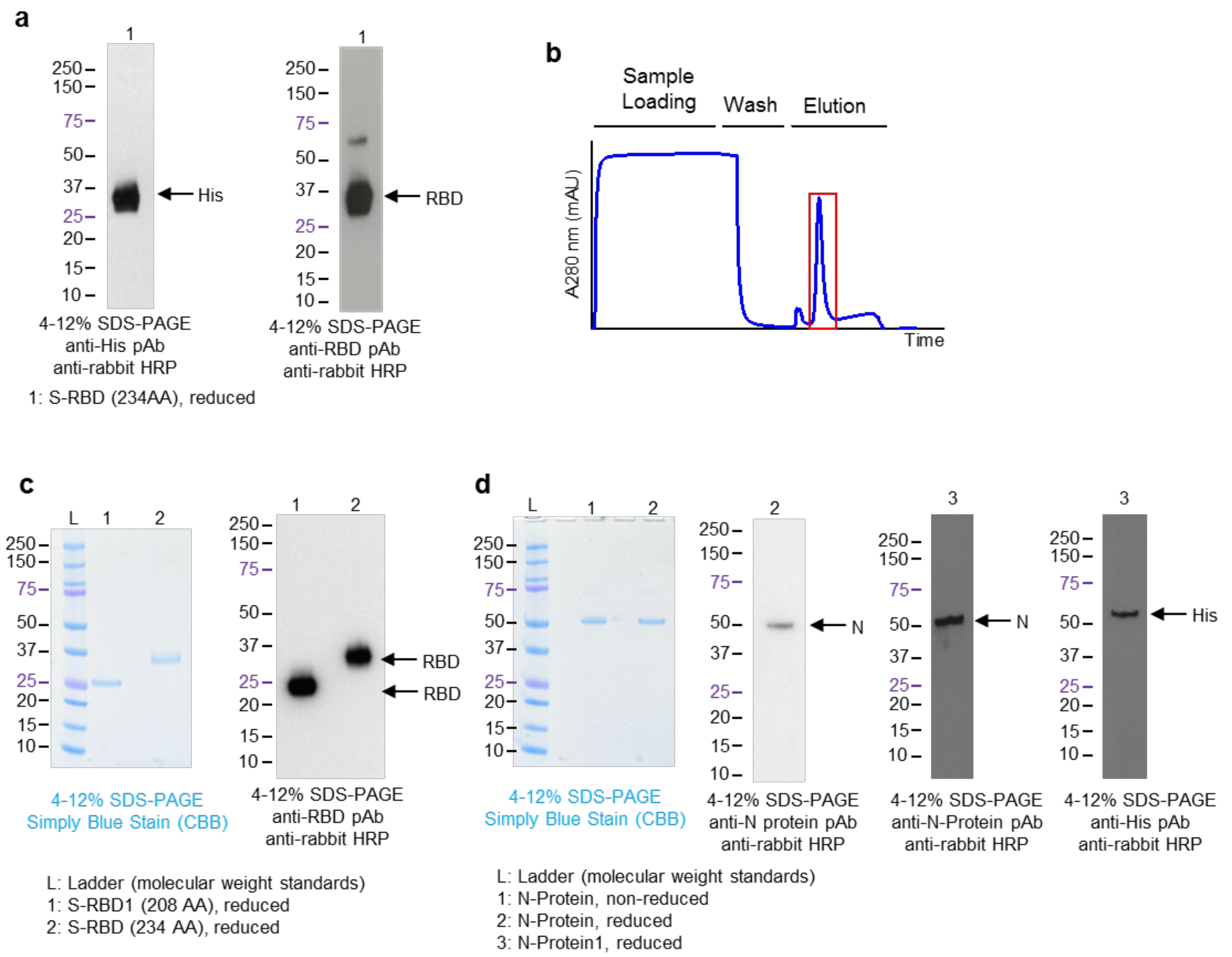

Figure S2: **Generation and validation of recombinant S-protein, S-RBD and N-protein**

(**a**) Representative Western blot for His-tag (left panel) and spike protein RBD (right panel) of S-protein and S-RBD. (**b**) Representative chromatogram of His-Trap isolated S-RBD (S-RBD1). (**c**) Representative Coomassie stain of S-RBD (left panel) and Western blot (right panel) for S-RBD and S-RBD1. (**d**) Representative Coomassie stain (left panel) of N-protein and Western blots for N-protein (second panel) and N-protein (third panel) and His-tag (right panel) of N-protein1.

Figure S3

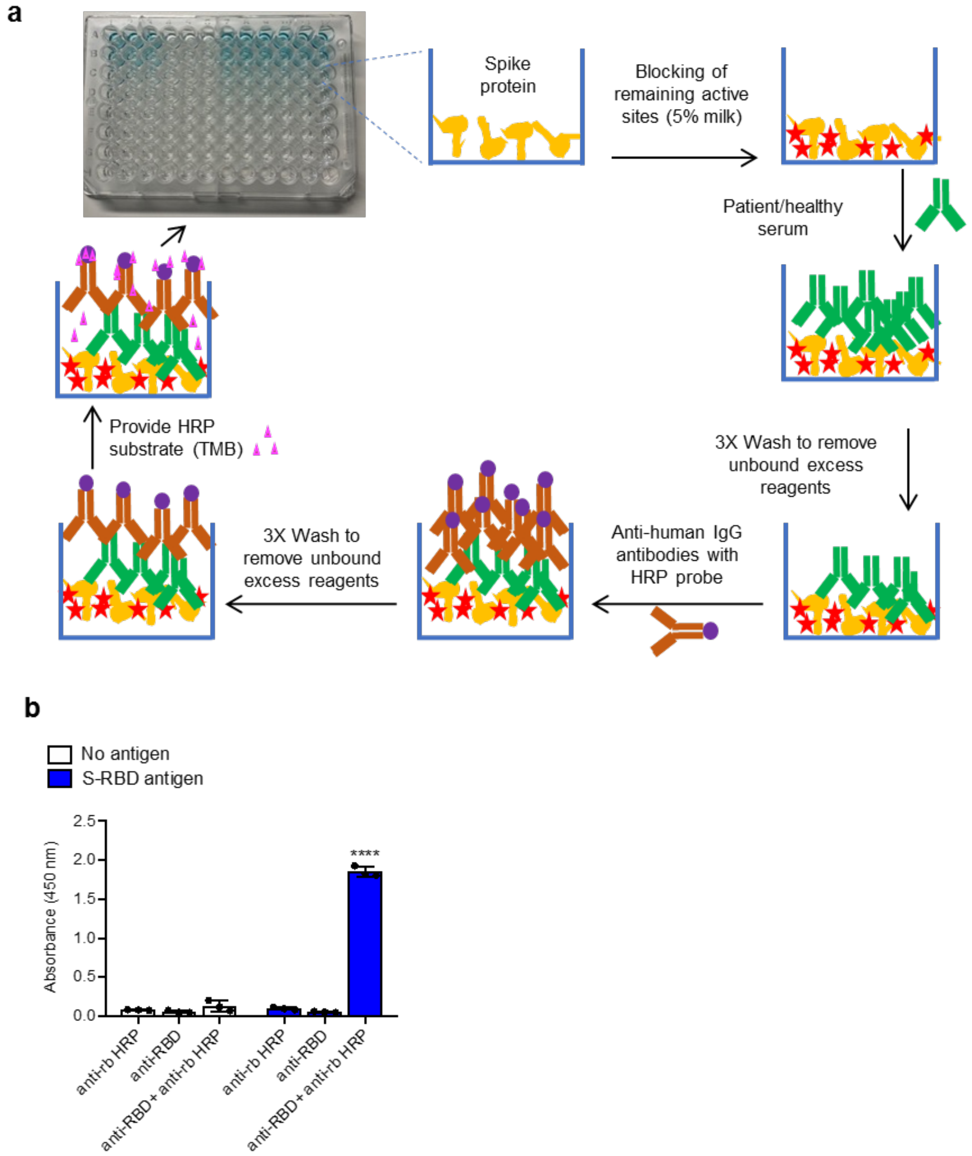

Figure S3: Design of ELISA-based serology test

(**a**) Schematic of ELISA protocol. Spike protein, orange; blocking reagent, red; primary antibody/serum, green; secondary antibody, brown; horseradish peroxidase (HRP), purple; HRP substrate, magenta. (**b**) ELISA with S-RBD antigen coating (2.5 μg/mL, 93.28 nM) and probed with anti-RBD antibody. Absorbance at 450 nm reported. Data is reported as mean ± standard deviation (s.d.) of 3 technical replicates for each sample. One-way ANOVA with Dunnett's multiple comparison test to no antigen+anti-rb HRP, **** $P < 0.0001$.

Figure S4

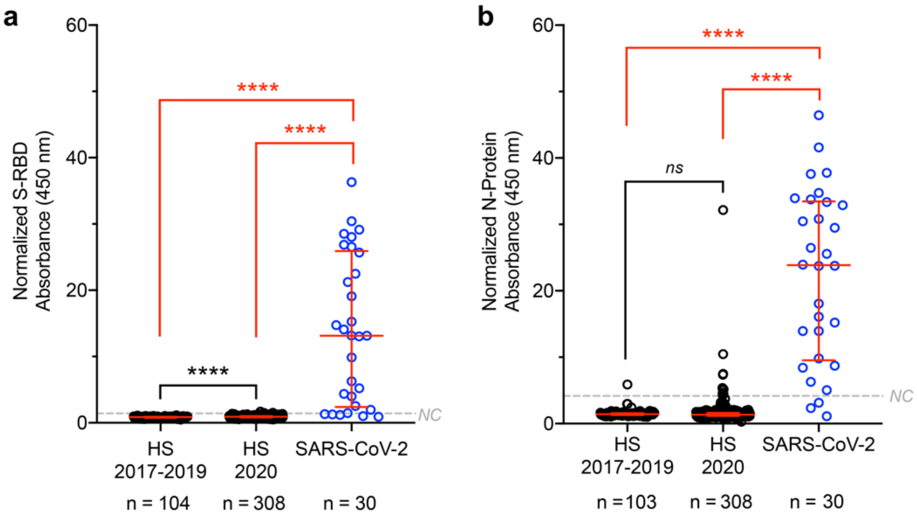

c

	N SARS-CoV-2	N HS 2017-2019	α error probability	Effect size	Power
S-RBD, CP included	30	104	0.025	1.1749	0.9994
S-RBD, CP excluded	21	104	0.025	0.9700	0.9533
N-Protein, CP included	30	103	0.025	1.6037	0.9999
N-Protein, CP excluded	21	103	0.025	1.3063	0.9988

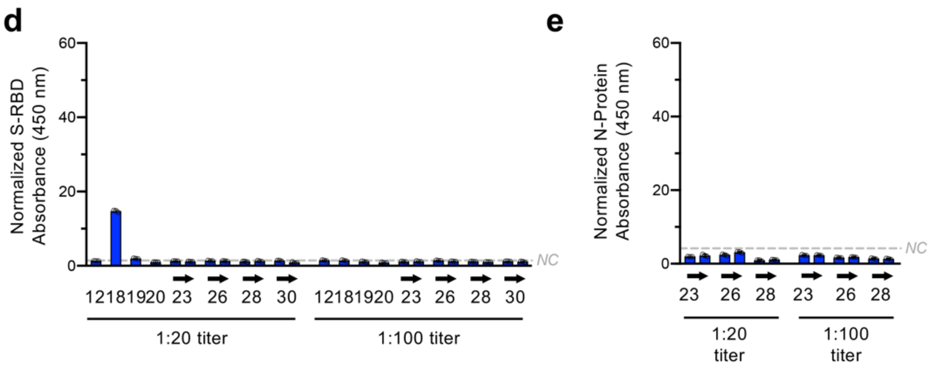

Figure S4: Statistical impact of convalescent plasma treatment and detection of low titer antibodies

(**a-b**) Absorbance normalized to the respective no antigen control for each sample at 450 nm plotted for S-RBD (**a**), and N-protein (**b**) antigen coating with the most recent (or only) serum sample from all SARS-CoV-2 patients and healthy samples separated based on time period of

serum collection. Data is presented with each dot representing the mean normalized absorbance for a given serum sample, the red bar depicts the median ± interquartile range of all samples. HS, healthy samples; NC, negative control cutoff, see methods. SARS-CoV-2, n=30; HS 2017-2019, n=104 for a, n=103 for b; HS 2020, n-308. Kruskall-Wallis with Dunn's multiple comparison test performed. **** $P < 0.0001$, *ns*: not significant. (**c**) Power analysis of normalized absorbance for each antigen with convalescent plasma samples included (CP included) or excluded (CP excluded). CP: convalescent plasma. Post-hoc Wilcoxon-Mann-Whitney test performed with Bonferroni-corrected α error probability (α=0.05/2 to account for two antigens). (**d**) ELISA with S-RBD protein coating and 1:20 dilution of serum samples of SARS-CoV-2 patients. Absorbance normalized to the respective no antigen control for each sample at 450 nm reported. SARS-CoV-2, n=12 serum samples (first or only and last sample from 8 patients). (**e**) ELISA with N-protein coating and 1:20 dilution of serum samples of SARS-CoV-2 patients. Absorbance normalized to the respective no antigen control for each sample at 450 nm reported. SARS-CoV-2, n=6 serum samples from 3 patients. Arrows lists consecutive serum samples evaluated for each case; data is reported as mean ± standard deviation (s.d.) of 3 technical replicates for each sample. NC, negative control cutoff, see methods.

S-RBD

Figure S5

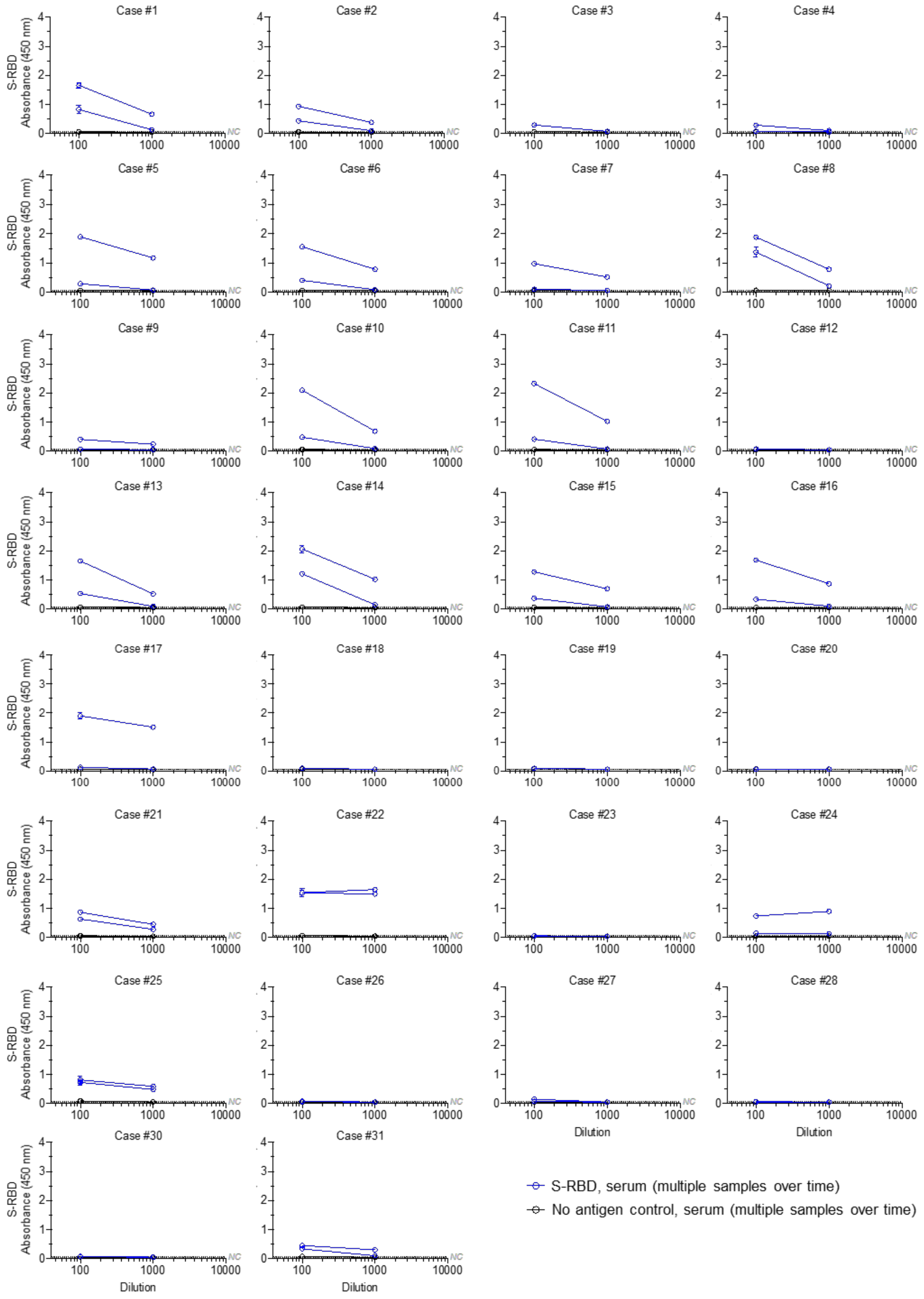

Figure S5: **Individual representation of serum dilutions (titers) tested for binding antibodies against S-RBD**

ELISA with S-RBD protein coating and serial dilutions of serum. Fold dilution of serum indicated. Each line represents one serum sample from a given patient. Absorbance at 450 nm reported. S-RBD antigen (blue), no antigen control (black) with SARS-CoV-2 serum. n=30 SARS-CoV-2^{+} patients with multiple serum samples. Data is reported as mean ± standard deviation (s.d.) of 3 technical replicates for each serum sample dilution. NC, negative control (secondary antibody without serum).

Figure S6

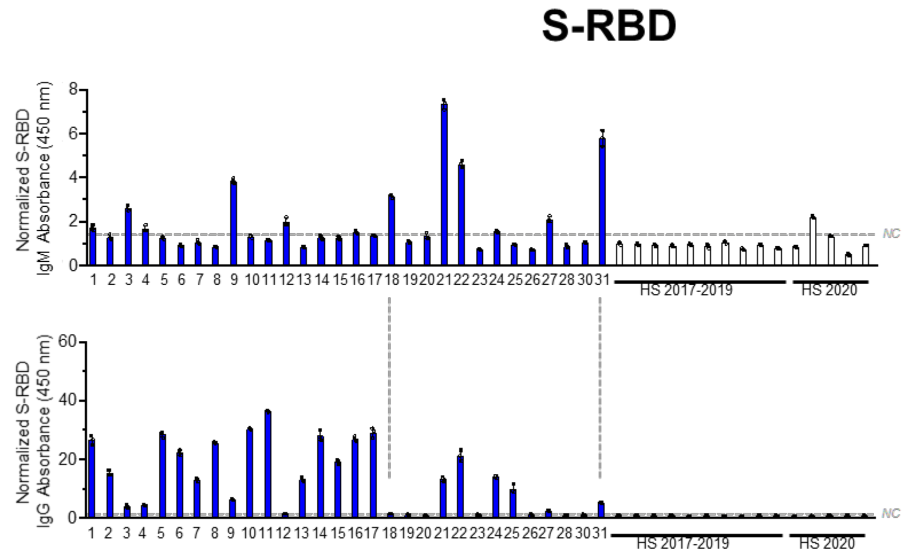

Figure S6: **Detection of IgM antibodies against S-RBD**

IgM (top panel) and IgG (bottom panel) ELISA with S-RBD protein coating and 1:100 dilution of the last serum sample from SARS-CoV-2 patients and healthy individuals. SARS-CoV-2 (blue), n=30; healthy samples from 2017-2019 (HS 2017-2019, white), n=10; healthy samples from 2020 (HS 2020, white), n=5. Absorbance normalized to the respective no antigen control for each sample at 450 nm reported. Data is reported as mean ± standard deviation (s.d.) of 3 technical replicates for each sample. NC, negative control cutoff (see methods).

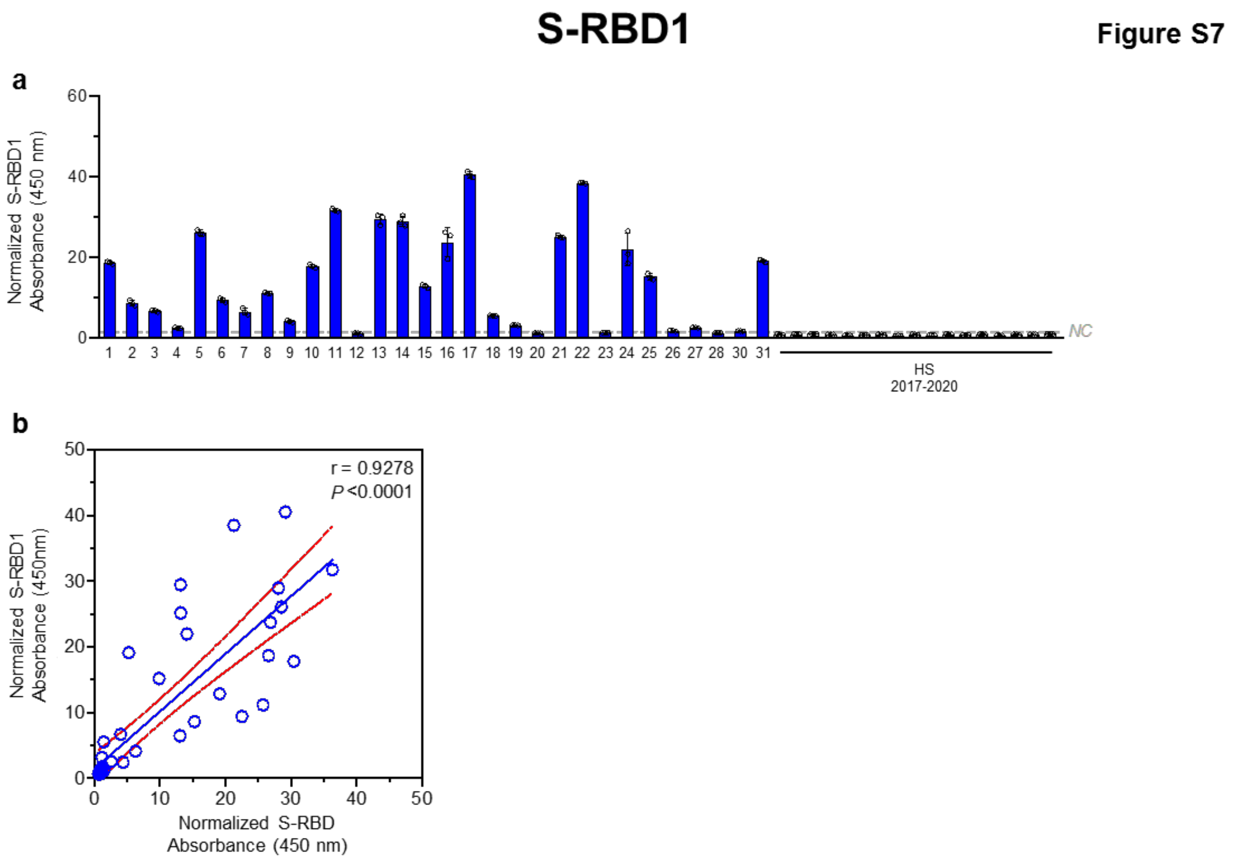

Figure S7: Detection of IgG antibodies against S-RBD1

(**a**) ELISA with S-RBD1 protein coating and 1:100 dilution of serum from SARS-CoV-2 patients and healthy individuals. SARS-CoV-2 (blue), n=30; healthy samples from 2017-2019 (HS 2017-2019, white), n=12; healthy samples from 2020 (HS 2020, white), n=5. Absorbance normalized to the respective no antigen control for each sample at 450 nm reported. Data is reported as mean ± standard deviation (s.d.) of 3 technical replicates for each sample. NC, negative control cutoff (see methods). (**b**) Correlation between S-RBD and S-RBD1 normalized absorbance. Spearman correlation coefficient (r) and *P* value reported.

N-Protein

Figure S8

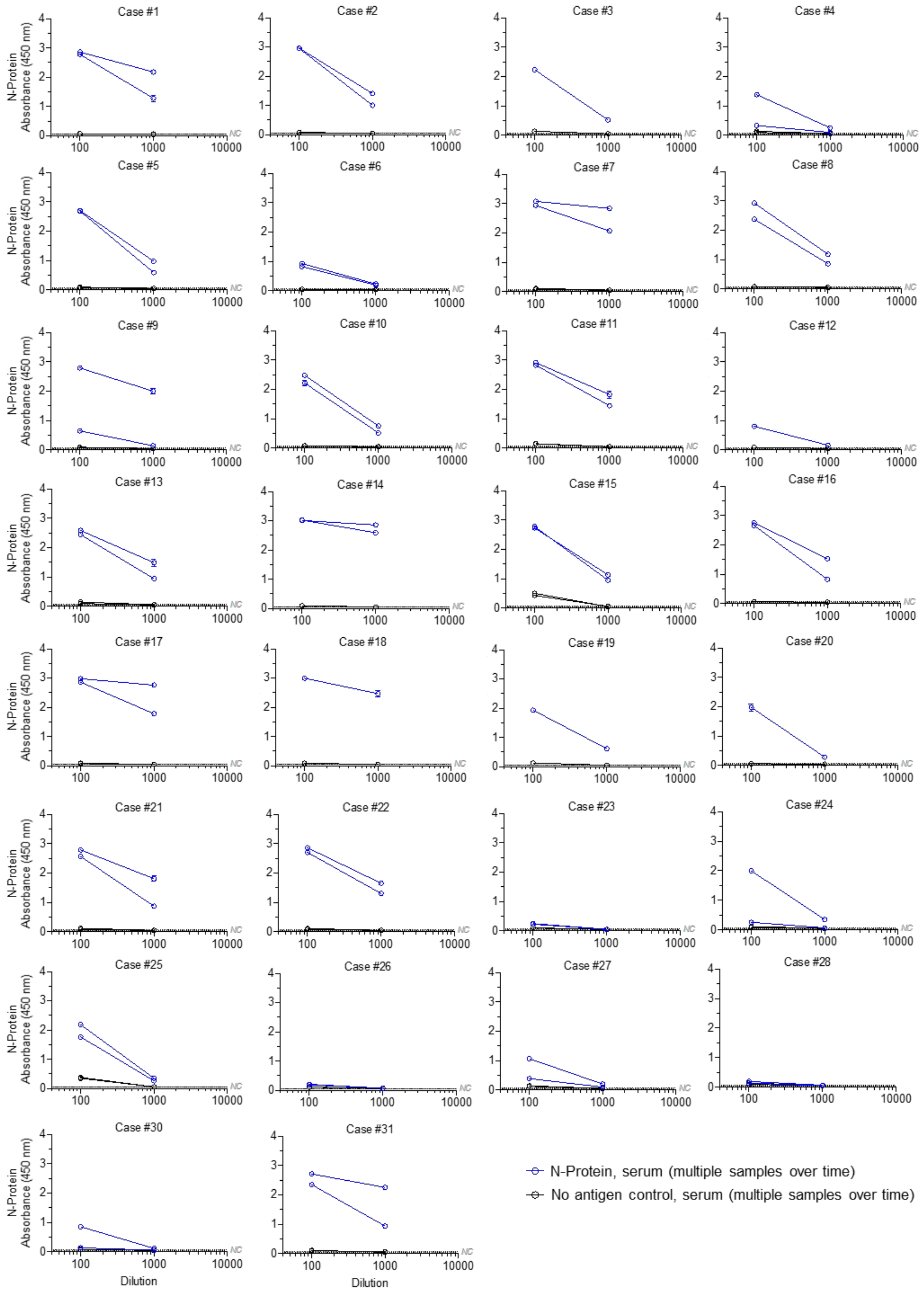

Figure S8: **Individual representation of serum dilutions (titers) tested for binding antibodies against N-protein**

ELISA with N-protein coating and serial dilutions of serum. Fold dilution of serum indicated. Each line represents one serum sample from a given patient. Absorbance at 450 nm reported. N-protein antigen (blue), no antigen control (black) with SARS-CoV-2 serum. n=30 SARS-CoV-2$^+$ patients with the first and most recent serum samples per patient analyzed. Data is reported as mean ± standard deviation (s.d.) of 3 technical replicates for each serum sample dilution. NC, negative control (secondary antibody without serum).

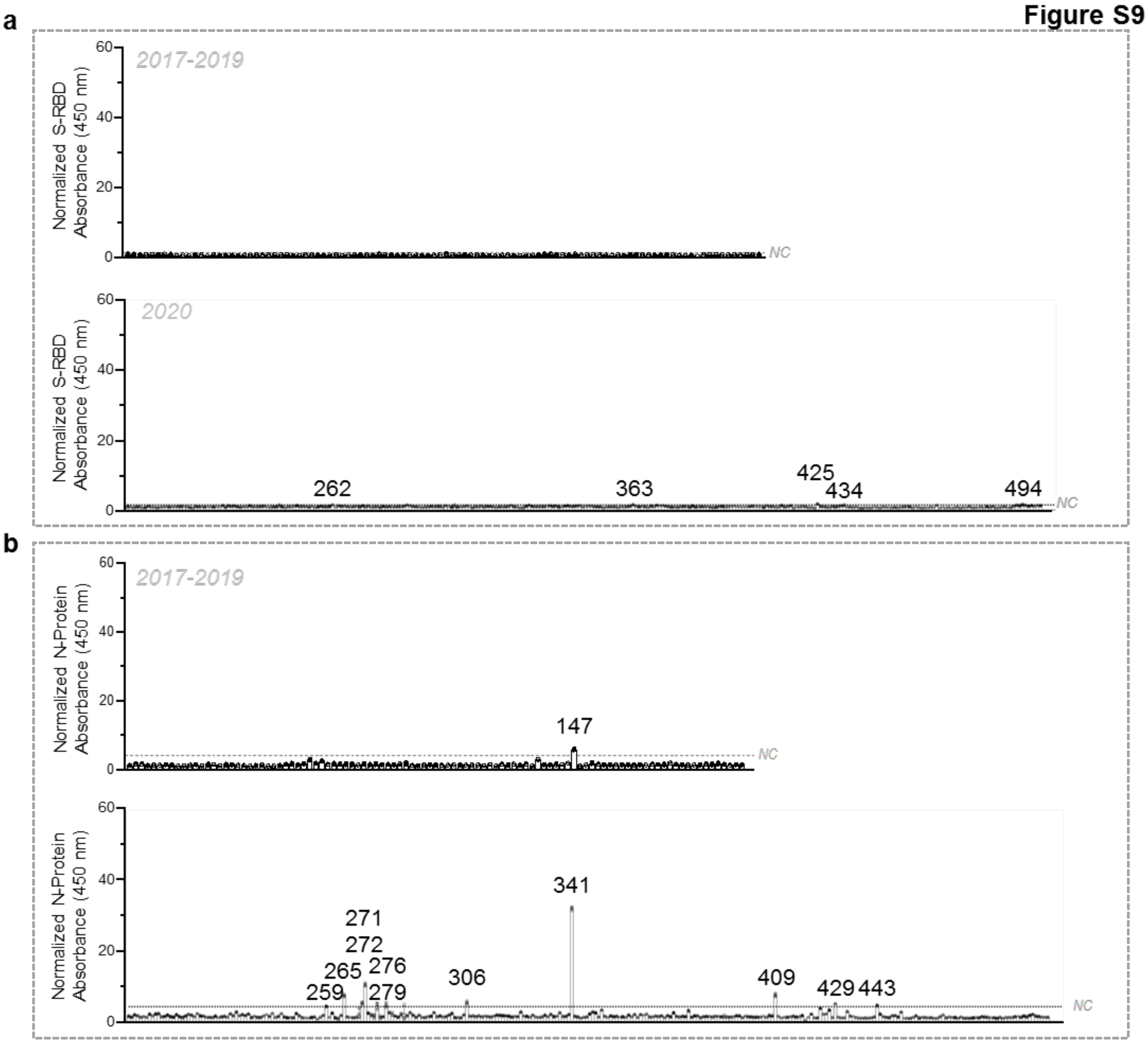

Figure S9: **Detailed collective representation of healthy serum dilution (titers) tested for binding antibodies**

(**a**) ELISA with S-RBD protein coating and 1:100 dilution of serum from healthy individuals from 2017-2019 (top panel) and 2020 (bottom panel). Samples with positive signal are denoted with their respective sample ID. Absorbance normalized to the respective no antigen control for each sample at 450 nm reported. HS 2017-2019, n=104; HS 2020, n=308. (**b**) ELISA with N-protein coating and 1:100 dilution of serum from healthy individuals from 2017-2019 (top panel) and 2020 (bottom panel). Samples with positive signal are denoted with their respective sample ID.

Absorbance normalized to the respective no antigen control for each sample at 450 nm reported. HS 2017-2019, n=103; HS 2020, n=308. NC, negative control cutoff (see methods). Data in this figure are also shown in Figure 2.

Figure S10

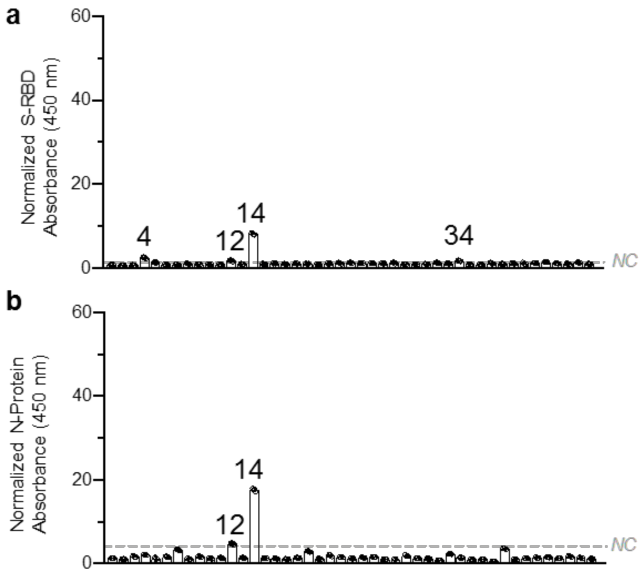

Figure S10: **Detailed collective representation of non-COVID-19 serum sample (NCS) dilution (titers) tested for binding antibodies**

(**a**) ELISA with S-RBD protein coating and 1:100 dilution of NCS serum. Samples with positive signal are denoted with their respective sample ID. Absorbance normalized to the respective no antigen control for each sample at 450 nm reported. n=45 NCS. (**b**) ELISA with N-protein coating and 1:100 dilution of serum from NCS. Samples with positive signal are denoted with their respective sample ID. Absorbance normalized to the respective no antigen control for each sample at 450 nm reported. n=45 NCS. NC, negative control cutoff (see methods).

Figure S11

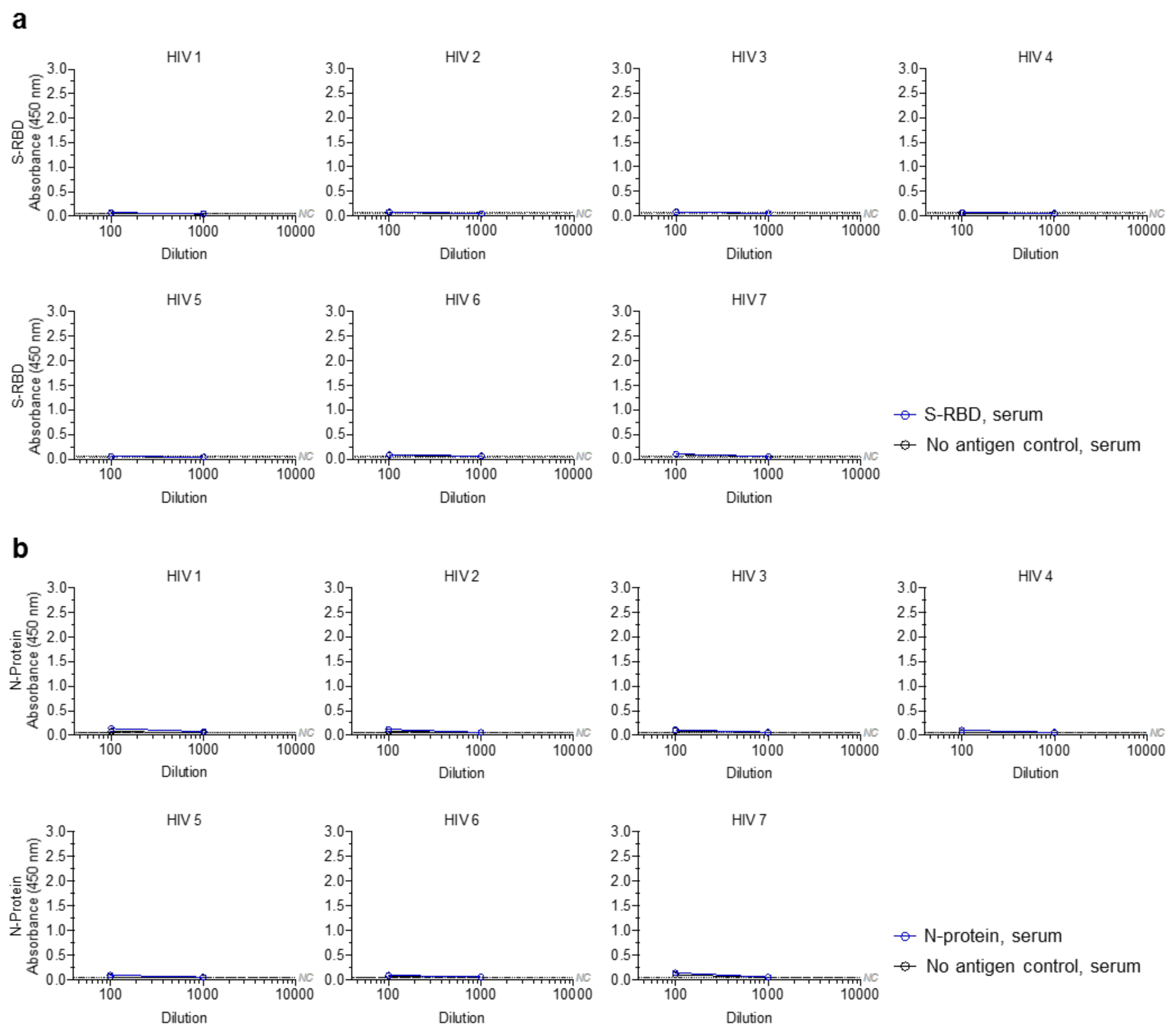

Figure S11: **Individual representation of cross-reactivity of HIV serum to SARS-CoV-2 antigens**

(**a**) ELISA with S-RBD protein coating and indicated dilutions of serum from HIV patients. (**b**) ELISA with N-protein coating and indicated dilutions of serum from HIV patients. Absorbance at 450 nm reported. S-RBD/N-protein antigen (blue), no antigen control (black) with HIV serum. n=7 HIV serum samples. Data is reported as mean ± standard deviation (s.d.) of 3 technical replicates for each serum sample dilution. NC, negative control (secondary antibody without serum).

Figure S12

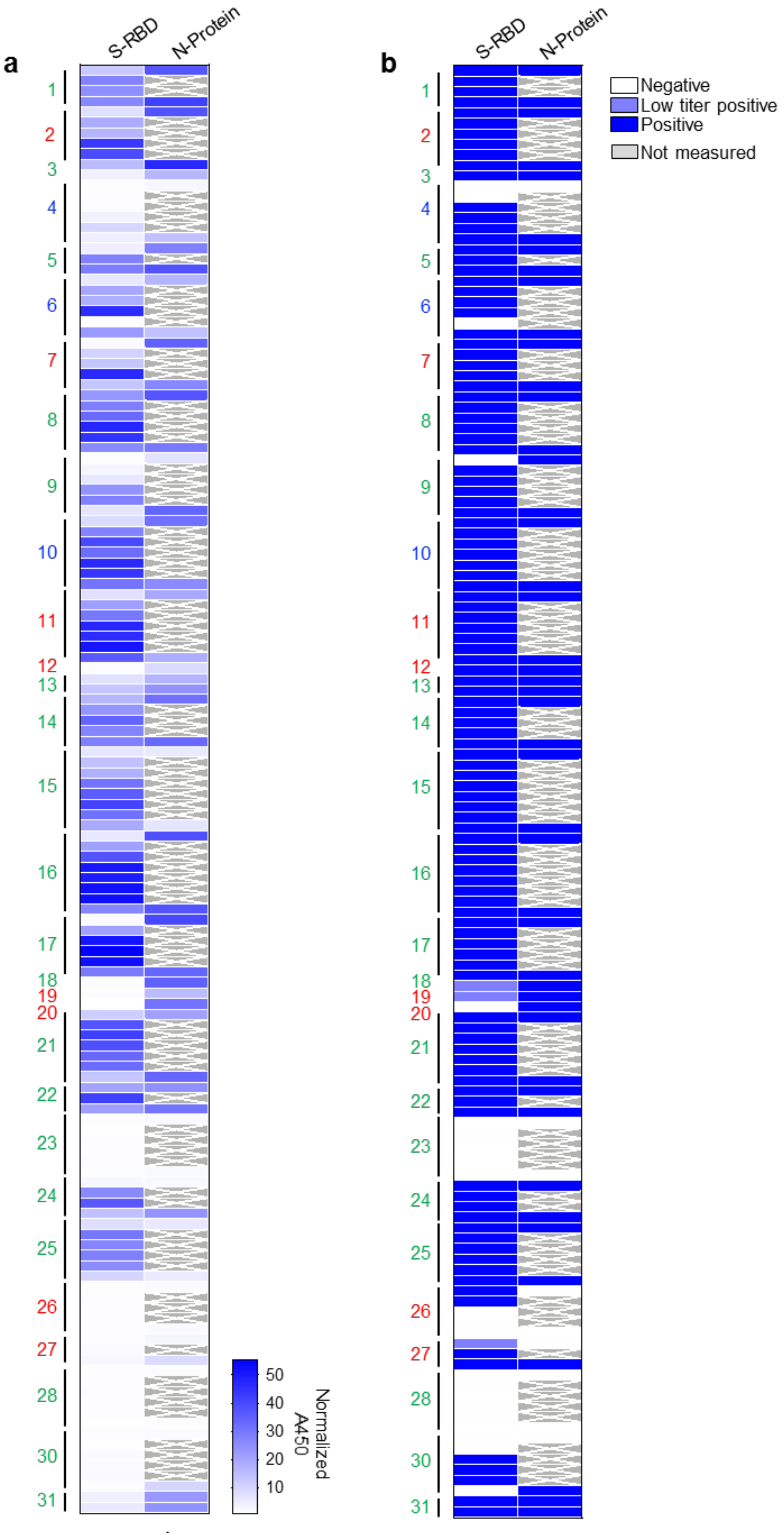
a
S-RBD
N-Protein
1
2
3
4
5
6
7
8
9
10
11
12
13
14
15
16
17
18
19
20
21
22
23
24
25
26
27
28
30
31
50
40
30
20
10
Normalized A450
b
S-RBD
N-Protein
Negative
Low titer positive
Positive
Not measured

Figure S12: **Comparative analyses of SARS-CoV-2 seroconversion to different viral antigens**

(**a**) Heatmap of normalized A450 values for each serum sample of each SARS-CoV-2 patients in the indicated serological tests. Absorbance normalized to the respective no antigen control for each sample at 450 nm reported. (**b**) Heatmap of positive, negative, and low titer positive categorization for each serum sample of each SARS-CoV-2 patients in the indicated serological tests. Case numbers are color coded, green: recovered, blue: hospitalized, red: deceased. LTP: low titer positive as defied by detecting of binding antibodies to shown in Figure S4d-e, 1:20 titer.

Figure S13

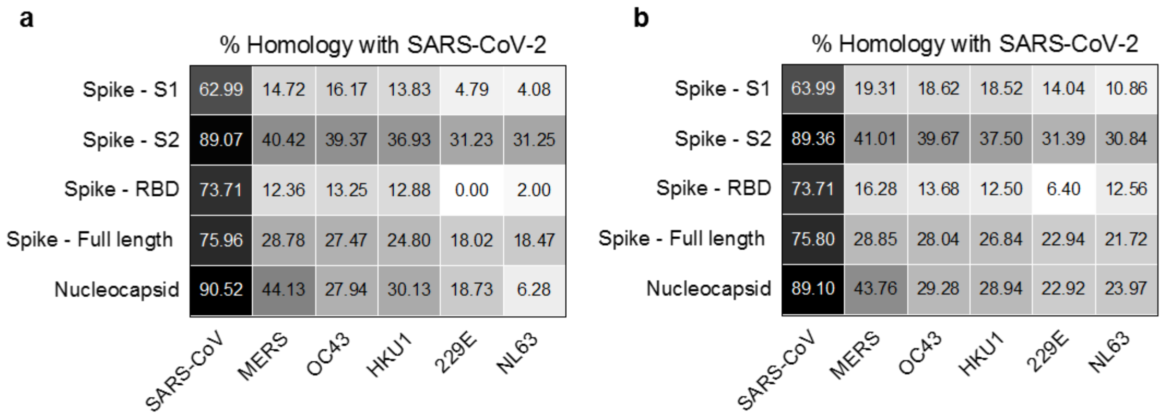

Figure S13: **Homology of spike and nucleocapsid proteins across coronaviruses**

(**a**) Percent homology for the listed proteins and coronaviruses using NCI BLASTp. (**b**) Percent homology for the listed proteins and coronaviruses using UniProt.

Figure S14

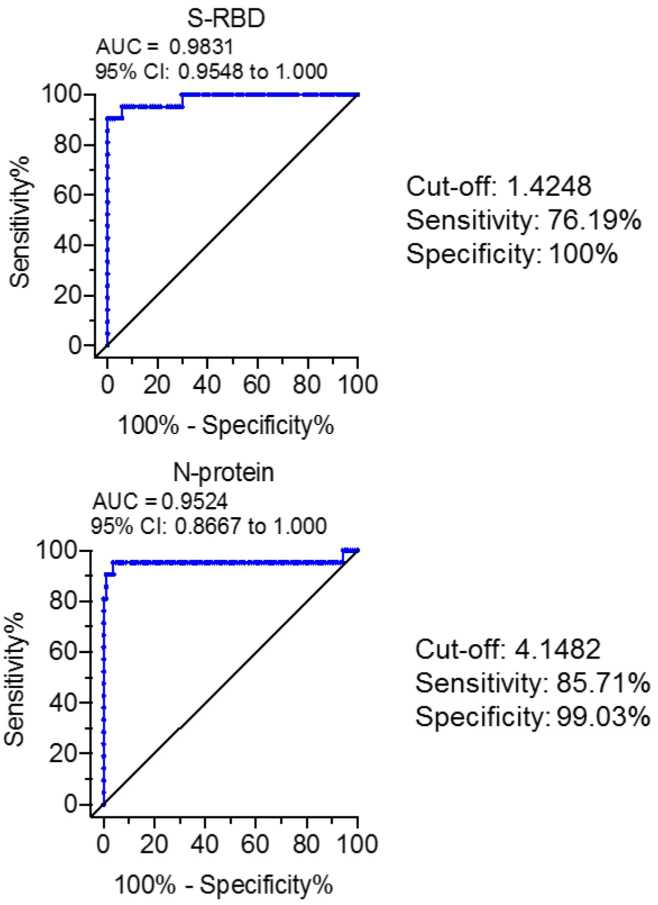

Figure S14: **Receiver operating characteristics for detection of seroconversion**

Receiver operating characteristics for S-RBD and N-protein ELISAs using n=21 SARS-CoV-2 serum (last or only available sample from patients not treated with convalescent plasma) and n=104 (S-RBD) or n=103 (N-protein) healthy serum samples from 2017-2019 (see methods). Cut-offs and associated specificity and sensitivity listed. AUC, area under the curve; CI: confidence interval.

Figure S15

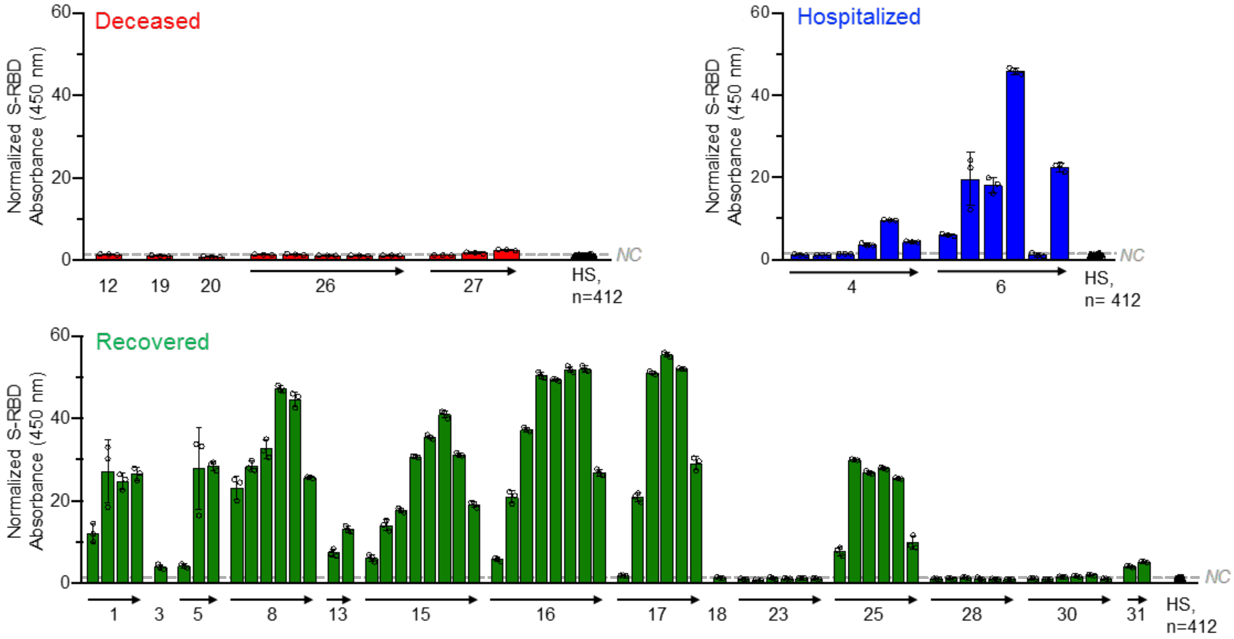

Figure S15: **Comparative analyses of SARS-CoV-2 seroconversion against different viral antigens, categorized based on clinical outcome**

Detailed representation (multiple serum samples) of data presented in Figure 3c. Arrows lists consecutive serum samples evaluated for each case.

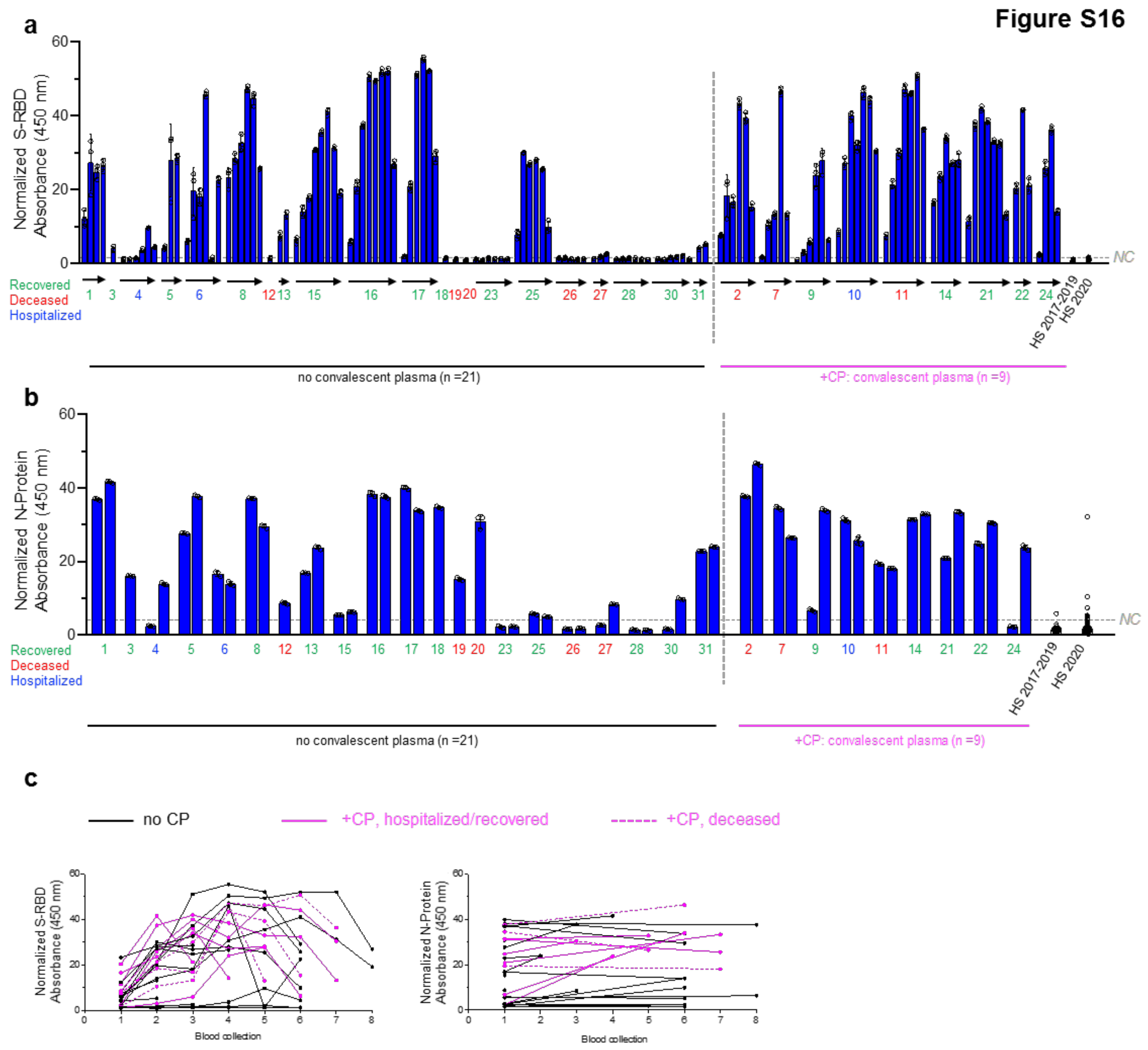

Figure S16: Circulating antibodies in COVID-19 patients infused with convalescent plasma

(**a**) ELISA with S-RBD protein coating and 1:100 dilution of repeated serum samples of SARS-CoV-2 patients and healthy individuals. Absorbance normalized to the respective no antigen control for each sample at 450 nm reported. SARS-CoV-2 (blue), n=138 (from 30 patients); healthy samples from 2017-2019 (HS 2017-2019, white), n=104; healthy samples from 2020 (HS 2020, white), n=308. (**b**) ELISA with N-protein coating and 1:100 dilution of the first and last serum samples of SARS-CoV-2 patients and healthy individuals. Absorbance normalized to the respective no antigen control for each sample at 450 nm reported. SARS-CoV-2 (blue), n=55

(from 30 patients); healthy samples from 2017-2019 (HS 2017-2019, white), n=103; healthy samples from 2020 (HS 2020, white), n=308. **a–b**: Arrows lists consecutive serum samples evaluated for each case; data is reported as mean ± standard deviation (s.d.) of 3 technical replicates for each sample. (**c**) Normalized A450 values over time (consecutive serum samples) for n=30 SARS-CoV-2 cases for the respective viral protein and subgroups (+CP: patient given convalescent plasma). NC, negative control cutoff, see methods; HS, healthy sample.

Figure S17

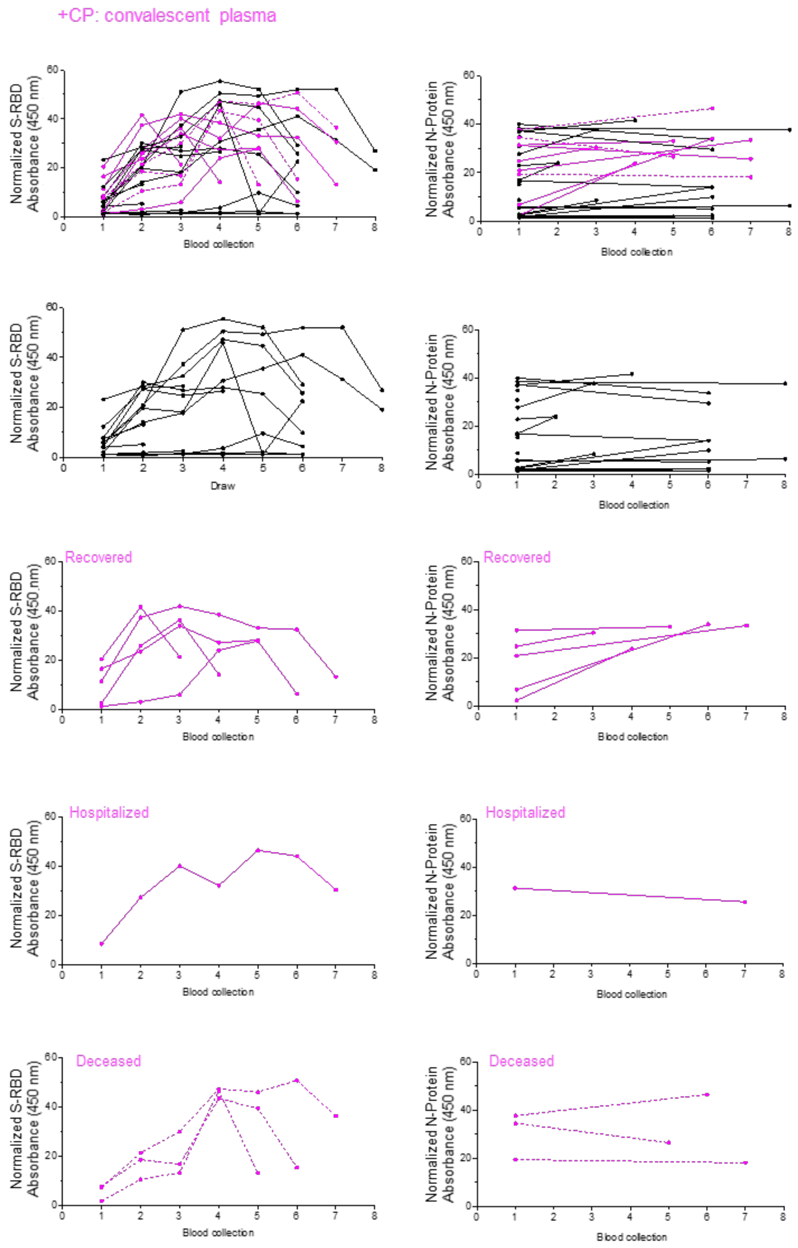

Figure S17: Detailed analysis of binding antibodies in COVID-19 patients treated with convalescent plasma.

Detailed representation (multiple serum samples) of data presented in Figure S16.

Figure S18

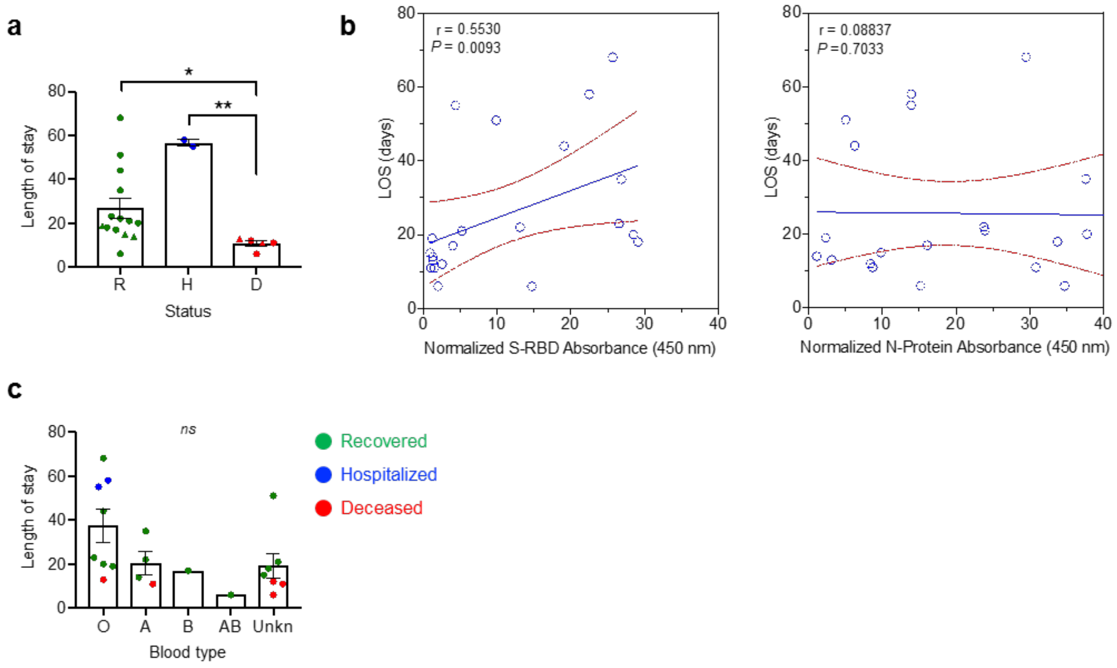

Figure S18: Analysis of the length of hospital stay, blood type, and seroconversion

(**a**) Representation of the length of stay (days) in SARS-CoV-2 cases based on outcome at the time of analysis. R: recovered, H: hospitalized, D: deceased. Triangles depict patients classified as S-RBD negative. (**b-c**) Correlation between length of stay (LOS) and normalized S-RBD absorbance (left panel) and normalized N-protein absorbance (right panel). Spearman correlation coefficient (r) and *P* value reported. (**c**) Length of stay of SARS-CoV-2 patients based on blood groups. Unkn: unknown blood type. Kruskall-Wallis with Dunn's multiple comparison test performed, * $P < 0.05$, ** $P < 0.01$, *ns*: not significant.

Figure S19

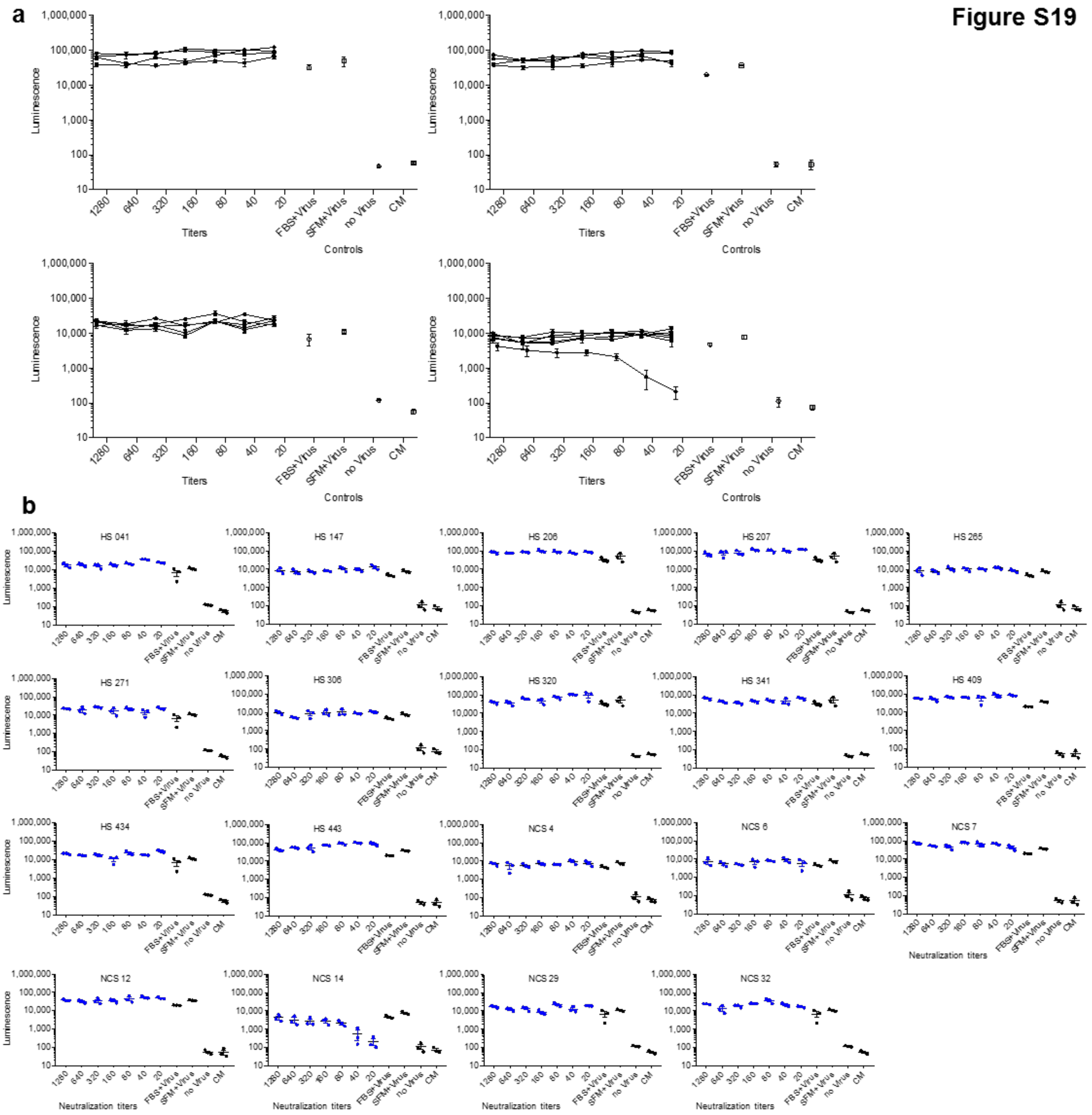

Figure S19: Detailed analysis of the neutralization activity of circulating antibodies in healthy and non-COVID-19 samples.

(**a**) Luminescence readings obtained from pseudovirus neutralization assay at the indicated serum dilutions for healthy (HS) and non-COVID-19 samples (NCS). Each graph depicts the distinct assays run and their respective controls. (**b**) Luminescence readings obtained from pseudovirus neutralization assay at the indicated titer. Data is reported as mean ± standard error

of the mean (s.e.m.) of 3 technical replicates. FBS+Virus, cells treated with virus in growth media containing fetal bovine serum (FBS); SFM, cells treated with virus in serum-free media (SFM); no virus, untreated cells; CM, control media (media removed).

Figure S20

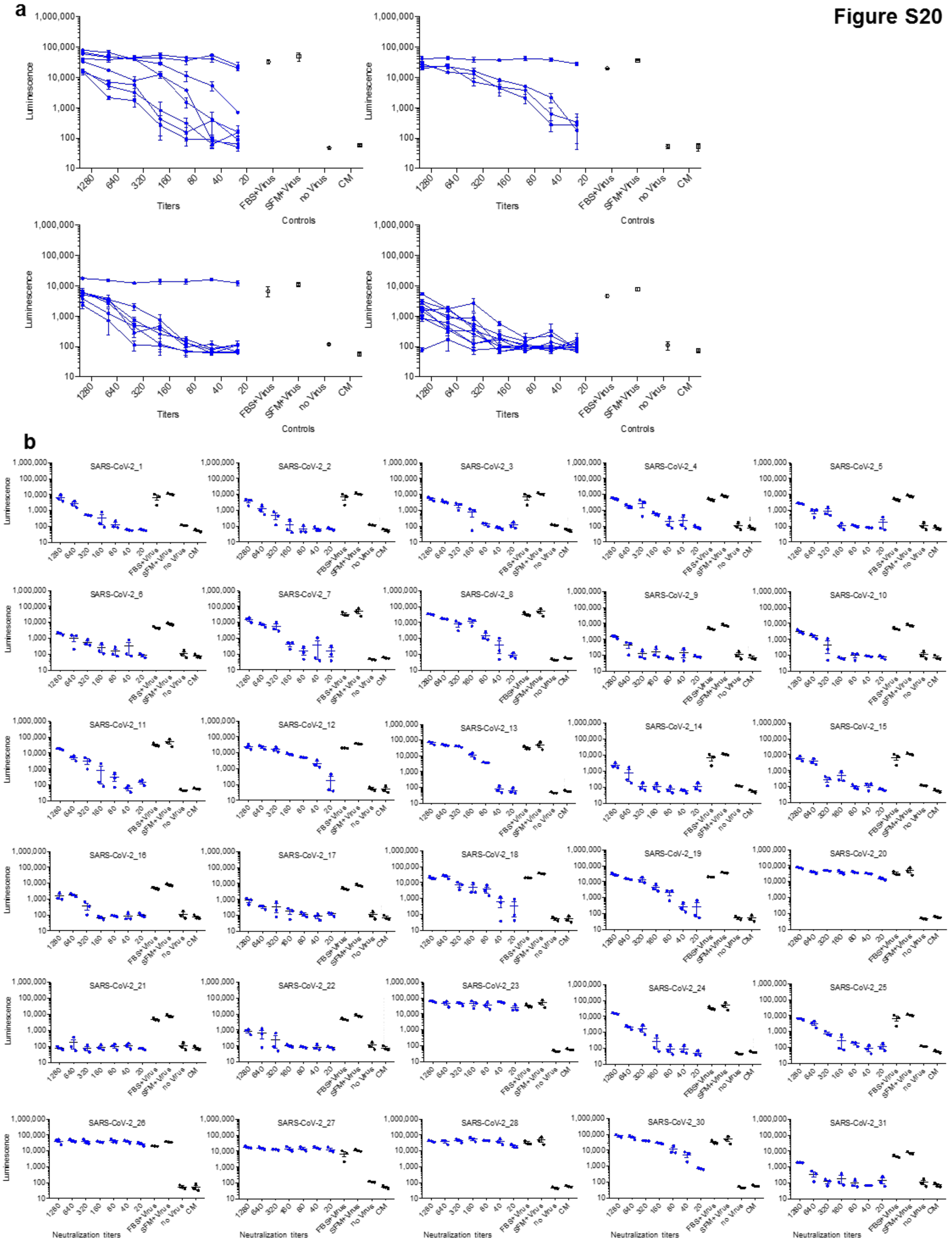

Figure S20: Detailed analysis of the neutralization activity of circulating antibodies in

SARS-CoV-2 patients.

(**a**) Luminescence readings obtained from pseudovirus neutralization assay at the indicated serum dilutions. Each graph depicts the distinct assays run and their respective controls. (**b**) Absolute values of luminescence obtained from pseudovirus neutralization assay at the indicated titer. Data is reported as mean ± standard error of the mean (s.e.m.) of 3 technical replicates. FBS+Virus, cells treated with virus in growth media containing fetal bovine serum (FBS); SFM, cells treated with virus in serum-free media (SFM); no virus, untreated cells; CM, control media (media removed).

Figure S21

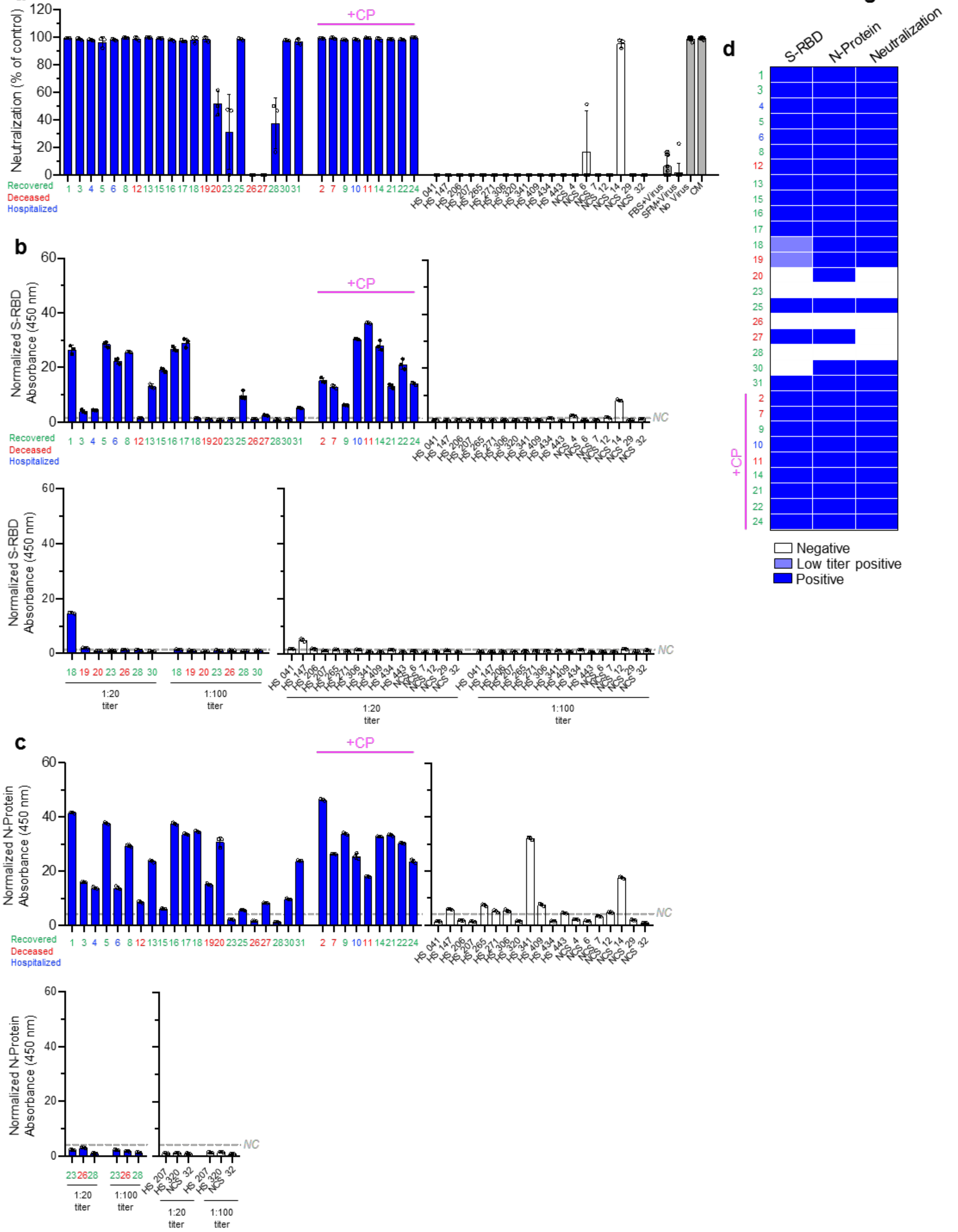

Figure S21: Pseudotyped SARS-CoV-2 virion neutralization activity of serum binding

antibodies to S-RBD and N-protein

(**a**) Luminescence normalized to FBS+virus control obtained from pseudovirus neutralization assay at 1:20 serum dilution (+CP: patient given convalescent plasma). (**b**) Matched serological results for S-RBD at 1:100 serum dilution (top two panels) and 1:20 serum dilution (bottom two panels). Absorbance normalized to the respective no antigen control for each sample at 450 nm reported. Case numbers are color coded, green: recovered, red: deceased, blue: hospitalized. (**c**) Matched serological results for N-protein at 1:100 serum dilution (top panel) and 1:20 serum dilution (bottom two panels). Absorbance normalized to the respective no antigen control for each sample at 450 nm reported. Case numbers are color coded, green: recovered, red: deceased, blue: hospitalized. Data (a-c) is reported as mean ± standard deviation (s.d.) of 3 technical replicates for each sample. (**d**) Heatmap depicting positive and negative categorization of the listed serum cases for each viral protein tested in serological and neutralization assays. Low titer positive as defied by detecting of binding antibodies to shown in Figure S4d-e, 1:20 titer.

Table S1: Reagents

**Recombinant proteins**	Vendor	Catalog
SARS-CoV-2 (2019-nCoV) spike RBD-His recombinant protein	Sino Biological	40592-V08H
SARS-CoV-2 (2019-nCoV) nucleocapsid-His recombinant protein	Sino Biological	40588-V08B

**Antibodies**	Vendor	Catalog	ELISA Dilution	WB Dilution
Strep TagII antibody	Sigma	71590-3	n/a	1:1000
His Tag antibody	Cell Signaling	2365S	n/a	1:1000
SARS-CoV-2 (2019-nCoV) spike RBD antibody	Sino Biological	40592-T62	1:5,000	1:1000
SARS-CoV-2 (2019-nCoV) nucleocapsid protein antibody	Sino Biological	40588-T62	n/a	1:2000
Goat anti-human IgG (Fc specific)-peroxidase	Sigma-Aldrich	A0170	1:10,000	n/a
Goat anti-human IgM mu chain (HRP)	Abcam	ab97205	1:20,000	n/a
Goat anti-mouse IgG (Fc specific)-peroxidase	Sigma-Aldrich	A0168	1:10,000	n/a
Goat anti-rabbit IgG (whole molecule)–peroxidase	Sigma-Aldrich	A0545	1:5,000	n/a
Goat anti-mouse IgG (H&L) HRP	Promega	W4021	n/a	1:2000
Donkey anti-rabbit IgG (H&L) HRP	Abcam	ab16284	n/a	1:2000

**Plasmids**	Vendor	Catalog
pcDNA3-SARS-CoV-2-S-RBD-8his (Spike-RBD-His)	Addgene	145145
SARS-CoV-2 (2019-nCoV) Nucleoprotein Gene ORF cDNA clone expression plasmid, C-His tag (N-Protein-His)	Sino Biological	VG40588-CH

**Reagents**	Vendor	Catalog
Sodium carbonate (Na2CO3)	Fisher Scientific	S495-500
Sodium bicarbonate (NaHCO3)	Sigma-Aldrich	S6014-25G
Hydrochoric acid (HCl)	Fisher Scientific	A144-212
Sodium hydroxide (NaOH)	Fisher Scientific	S318
Sulfuric acid (H2SO4)	Fluka	35276
Tris base	Fisher Scientific	BP152
PBS	Corning	21-040-CV
Sodium chloride (NaCl)	Fisher Scientific	BP358-1
Tween 20	Fisher Scientific	BP337-500
3,3′,5,5′-Tetramethylbenzidine reagent (TMB)	Thermo Fisher	34029
Bolt™ 4 to 12%, Bis-Tris, 1.0 mm, mini protein gel, 10-well	Invitrogen	NW04120BOX
Bolt™ 10%, Bis-Tris, 1.0 mm, mini protein gel, 12-well	Invitrogen	NW00102BOX
20X Bolt™ MES SDS running buffer	Invitrogen	B0002
4x Laemmli sample buffer	Bio-Rad	1610747
Trans-Blot turbo RTA mini 0.2 µm PVDF transfer kit	Bio-Rad	1704272
West-Q pico ECL solution	GenDEPOT	W3652-020
Amersham hyperfilm ECL	cytiva (formerly GE Healthcare)	28906835
SimplyBlue SafeStrain	Invitrogen	LC6060
EDTA-free protease inhibitor cocktail	Roche	04-693-159-001
Precison plus protein dual color standards	Bio-Rad	161-0374

**Protein Purification Reagents**	Vendor	Catalog
Qiagen Ni-NTA Superflow 5 mL	Qiagen	30761
StrepTrap HP 1 mL	cytiva (formerly GE Healthcare)	28907546
Amicon® Ultra-4 centrifugal filter unit	MilliporeSigma	UFC803024
Biotin	Sigma-Aldrich	B4501
Imidazole	Sigma-Aldrich	I5513
Freestyle 293 expression medium	Thermo Fisher	12338-018
FreeStyle™ 293-F cells	Thermo Fisher	R79007
293fectin reagent	Thermo Fisher	12347-019
Qubit protein assay kit	Thermo Fisher	Q33212

Full unedited gel for Figure S1c

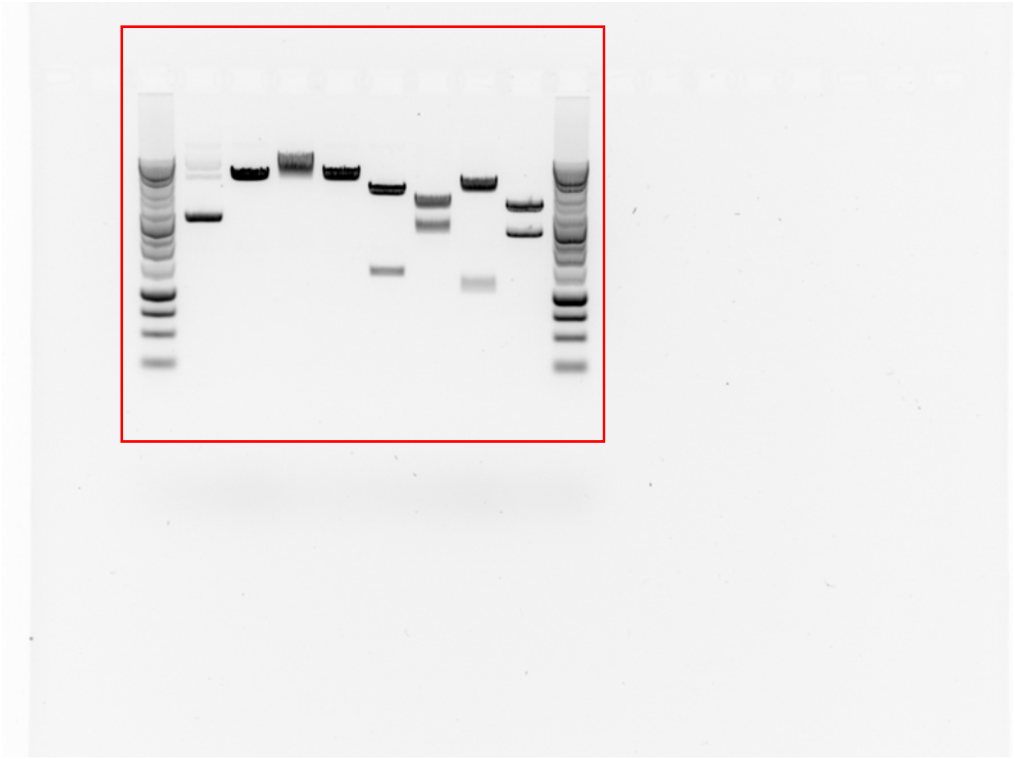

Full unedited gel for Figure S1f

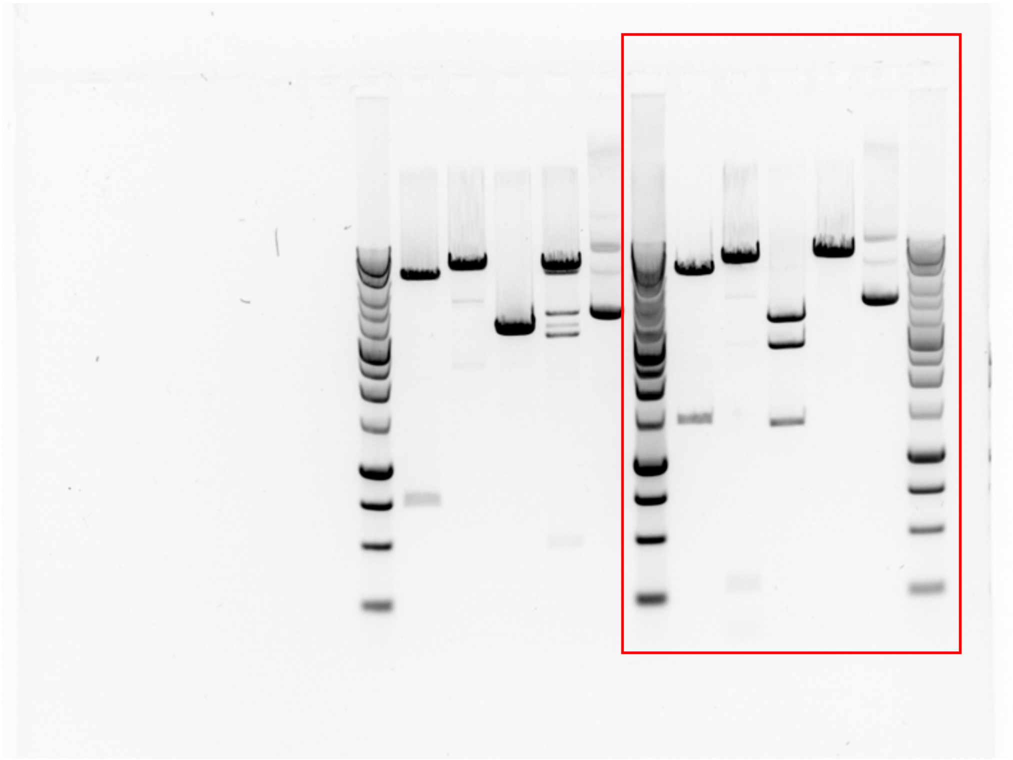

Full unedited blot for Figure S2a

Left panel
Anti-His

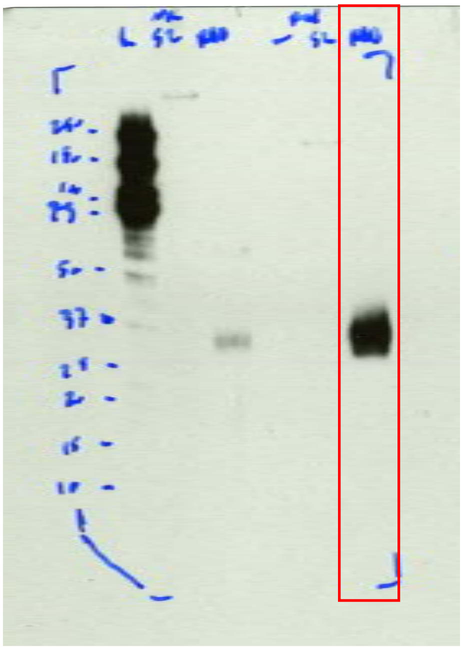

Right panel
Anti-RBD

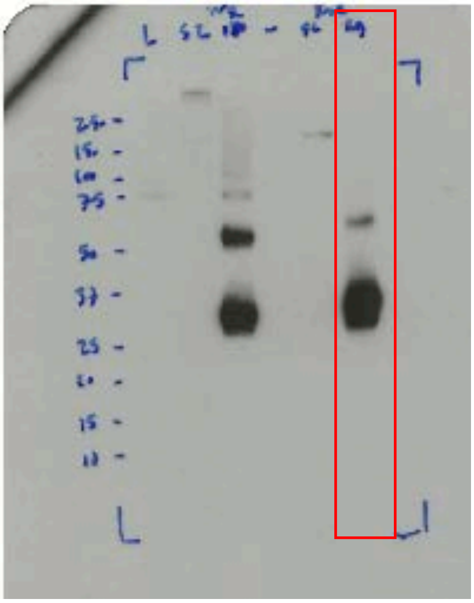

Full unedited gel/blot for Figure S2c

Left panel
Coomassie gel

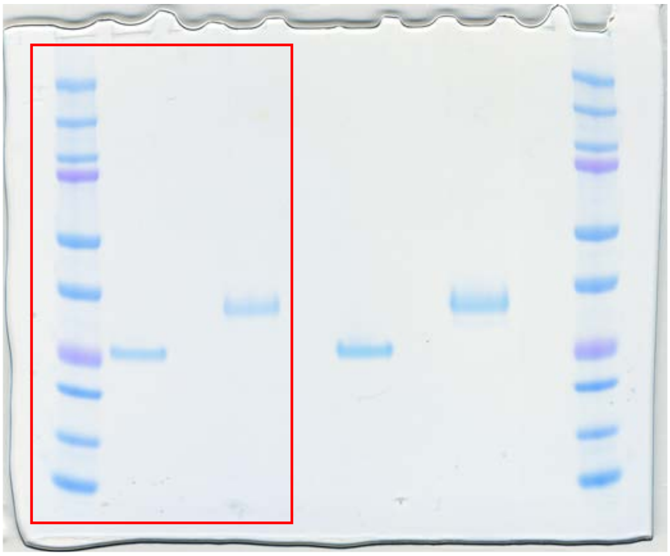

Right panel
Anti-RBD

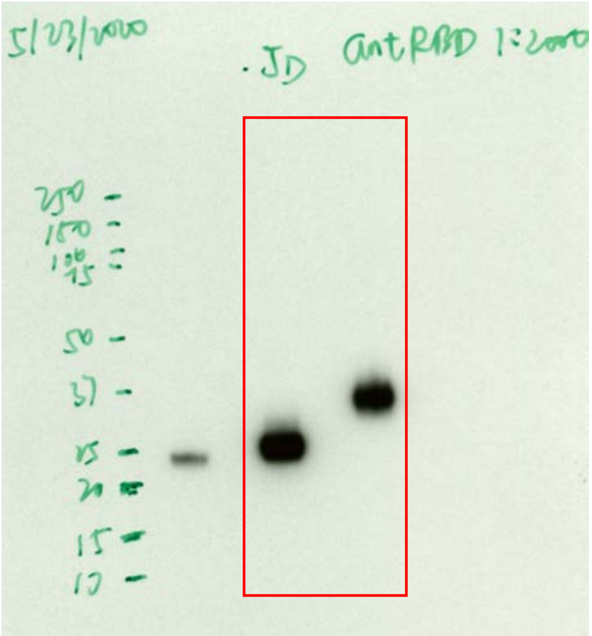

Full unedited gel/blots for Figure S2d

First panel
Coomassie gel

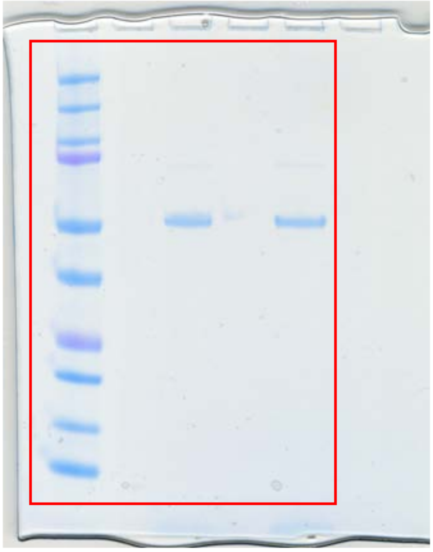

Second panel
Anti-N-protein

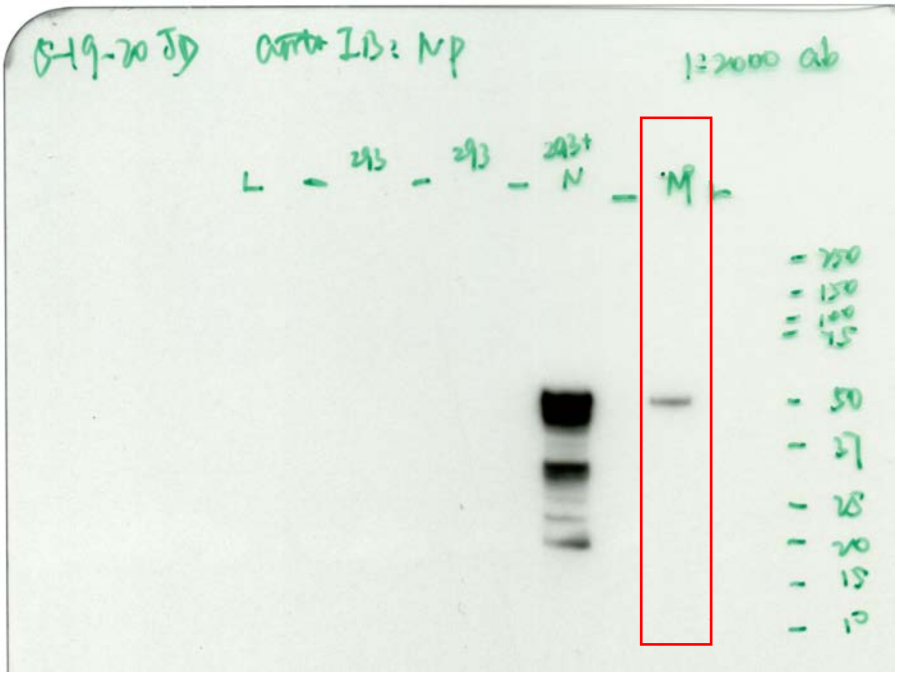

Third panel
Anti-N-protein

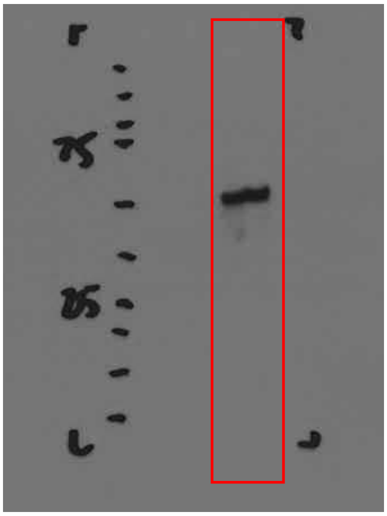

Fourth panel
Anti-His

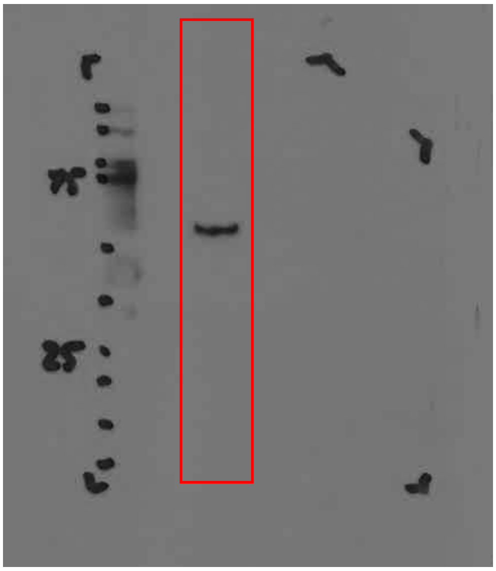

Supplement: Supplemental data [file jciinsight-5-142386-s109.pdf]
